# Comparison and interpretation of characteristics of Rhizosphere microbiomes of three blueberry varieties

**DOI:** 10.1186/s12866-021-02092-7

**Published:** 2021-01-22

**Authors:** Yan Zhang, Wei Wang, Zhangjun Shen, Jingjing Wang, Yajun Chen, Dong Wang, Gang Liu, Maozhen Han

**Affiliations:** 1 School of Life Sciences, Hefei Normal University, Hefei, 230601 Anhui China; 2 School of Life Sciences, Anhui Medical University, Hefei, 230032 Anhui China

**Keywords:** Rhizosphere microbial community, Rhizosphere microbiome, Blueberry, Biomarkers, Co-occurrence network

## Abstract

**Background:**

Studies on the rhizosphere microbiome of various plants proved that rhizosphere microbiota carries out various vital functions and can regulate the growth and improve the yield of plants. However, the rhizosphere microbiome of commercial blueberry was only reported by a few studies and remains elusive. Comparison and interpretation of the characteristics of the rhizosphere microbiome of blueberry are critical important to maintain its health.

**Results:**

In this study, a total of 20 rhizosphere soil samples, including 15 rhizosphere soil samples from three different blueberry varieties and five bulk soil samples, were sequenced with a high-throughput sequencing strategy. Based on these sequencing datasets, we profiled the taxonomical, functional, and phenotypic compositions of rhizosphere microbial communities for three different blueberry varieties and compared our results with a previous study focused on the rhizosphere microbiome of blueberry varieties. Our results demonstrated significant differences in alpha diversity and beta diversity of rhizosphere microbial communities of different blueberry varieties and bulk soil. The distribution patterns of taxonomical, functional, and phenotypic compositions of rhizosphere microbiome differ across the blueberry varieties. The rhizosphere microbial communities of three different blueberry varieties could be distinctly separated, and 28 discriminative biomarkers were selected to distinguish these three blueberry varieties. Core rhizosphere microbiota for blueberry was identified, and it contained 201 OTUs, which were mainly affiliated with *Proteobacteria*, *Actinobacteria*, and *Acidobacteria*. Moreover, the interactions between OTUs of blueberry rhizosphere microbial communities were explored by a co-occurrence network of OTUs from an ecological perspective.

**Conclusions:**

This pilot study explored the characteristics of blueberry’s rhizosphere microbial community, such as the beneficial microorganisms and core microbiome, and provided an integrative perspective on blueberry’s rhizosphere microbiome, which beneficial to blueberry health and production.

**Supplementary Information:**

The online version contains supplementary material available at 10.1186/s12866-021-02092-7.

## Background

The rhizosphere of plants harbors diverse microorganisms in the soil, which evolve alongside plants and environments and form an integral part of plants’ life cycle. Rhizosphere microbiota carries out various vital functions and plays a critical role in biogeochemical cycles involving soil formation and carbon cycling [1]. For example, many rhizosphere microorganisms provide nutrients to plants from soil [2] and prevent plants from being infested by pathogens [3]. The complex and dynamic interactions between plants and microbiota, especially between microorganisms, are related to plants’ growth [4, 5]. Hence, understanding the taxonomical and functional compositions of the rhizosphere microbial community is beneficial to plants’ growth and yield. In recent decades, many studies have been conducted to characterize rhizosphere microbiome in specific crop plant species, including rice [6], soybean [7], corn [8], barley [9], and wheat [10], and vegetable and fruit crops, including sugarcane [11], cucumber [12], grapevine [13] and citrus [14, 15]. A majority of these studies were performed through high-throughput sequencing of the microbial 16S rRNA to fully explore and characterize the role of microbiota in the rhizosphere microbial community. Several consistent trends and specific traits were demonstrated based on many studies on the rhizosphere microbiome of plants. For example, the number of bacteria affiliated with Alphaproteobacteria in various plants’ rhizosphere microbial communities increases [9, 16, 17]. However, the current studies on the rhizosphere microbiome are primarily on model plants, and relatively few studies related to blueberry have been carried out to explore the taxonomical and functional compositions of the blueberry rhizosphere microbial community [18], especially for the rhizosphere microbiome of different blueberry varieties [19].

Blueberries are perennial flowering plants known for their blue or purple berries. In taxonomy, the species of blueberry are classified into the *Vaccinium* genus. The commercial blueberries are all native to North America, and different kinds of blueberries were later introduced to Asia and Europe [20]. In recent years, numerous studies have investigated the effects of blueberry on consumer’s health based on their composition in flavonoids, polyphenols, anthocyanins, pro-anthocyanidins, phenolic acids and stilbenes, and demonstrated that the anti-oxidant and anti-inflammatory activities of blueberry [21, 22]. Moreover, previous studies have explored the dynamic changes of human or mice gut microbes with the consumption of blueberry or its extracts [23, 24]. Six-week regular consumption of wild blueberry drink can positively modulate the composition of human gut microbiota and increase the content of *Bifidobacteria* [23], which have been shown to exert positive benefits to humans health [25]. Additionally, growing evidence suggested that flavonoids of blueberry have the potential to restrict the development and severity of certain cancers and vascular diseases [26]. Given these benefits, more blueberries are needed and consumed. However, the diseases of blueberry, such as stem and leaf diseases, including phomopsis leaf spot and fruit rot and septoria leaf spot, reduce the yield of blueberry [27–29]. Previous studies have suggested that the rhizosphere microbiome can influence plant susceptibility to diseases and fitness response to environmental factors [30, 31], and several diseases of plants are related to rhizosphere microbiota in soil and can be controlled by related microbes [32, 33]. Therefore, understanding the blueberry rhizosphere microbiome and comparing the differences in rhizosphere microbial communities of different blueberry varieties, including the universal microbiota (shared microbiota) between different kinds of blueberry varieties and specific microbiota of each blueberry, are favorable to the cultivation and agricultural management of blueberries. However, only a few studies have explored and illustrated the composition of the rhizosphere microbiome of blueberry to date [19] and the composition of blueberry rhizosphere microbial community remains elusive.

In this present study, we collected 15 rhizosphere soil samples of three kinds of blueberry varieties, including Rabbiteye Blueberry (*Vaccinium virgatum*), Northern Highbush Blueberry (*V. corymbosum*), and Southern Highbush Blueberry (an interspecific hybrid of *V. corymbosum* and *V. darrowii*), and five adjacent soil samples (bulk soil) from a blueberry plantation in Hefei City, China, on 13 April 2018. To profile the structure of rhizosphere microbial community of blueberries, we performed 16S rRNA amplicon sequencing for these samples and analyzed the sequencing data. In this work, we focused on the following scientific questions: (i) How does the microbial diversity differ between rhizosphere microbial communities of different blueberry varieties? (ii) What are the differences in taxonomical, functional, and phenotypic compositions between rhizosphere microbial communities of different blueberry varieties? (iii) What is the core microbiota of rhizosphere microbial communities in blueberry? (iv) How are the co-occurrence relationships between the microbiota in different blueberry varieties? Notably, our study aims to compare and interpret the characterization of the blueberry rhizosphere microbial community and explore the patterns of the blueberry rhizosphere microbial community, which could provide an integrative view on the blueberry rhizosphere microbiome and provide insights on keeping blueberry health to improve the production of blueberry.

## Results

### Differential microbial diversity in blueberry rhizosphere microbial community

To profile the taxonomical compositions of rhizosphere microbial communities of blueberry and compare the taxonomical differences for these three blueberry varieties and bulk soil samples, we sequenced the V3–V4 region of 16S rDNA of bacteria and archaea from rhizosphere samples. In total, 997,713 high-quality 16S rRNA amplicons for 20 rhizosphere samples were obtained and analyzed. The number of sequences for these samples ranged from 31,591 to 73,918, with an average value 49,886 (Supplementary Table 1). After rarefying the final OTU table to 18,652 reads, we detected 6280 OTUs for these rhizosphere soil samples, and the number of OTUs for blueberry rhizosphere microbial communities and bulk samples ranged from 1495 OTUs to 2548 OTUs (Supplementary Table 1).

**Fig. 1:**
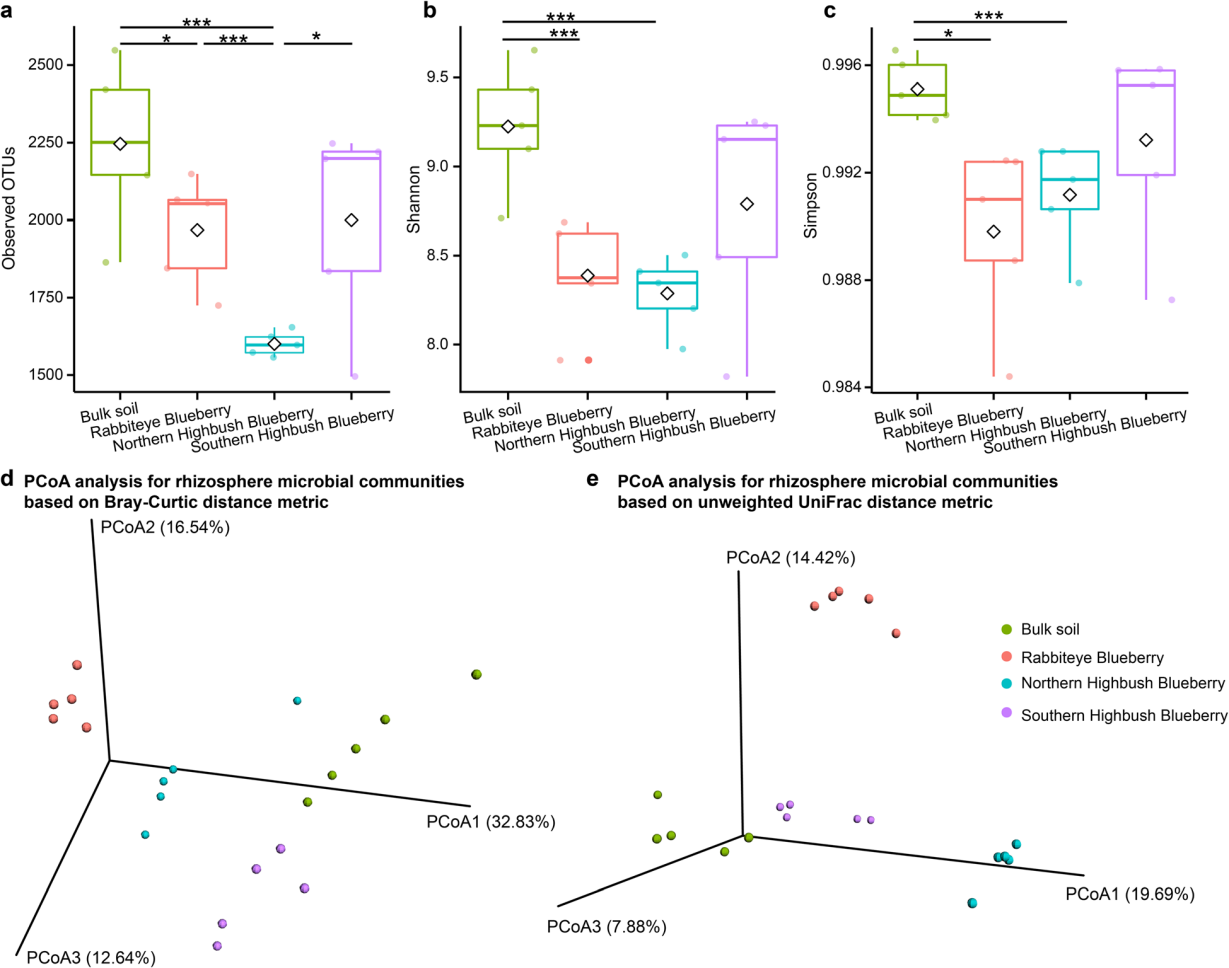
Microbial diversity of rhizosphere microbiota in three blueberry varieties. Comparison of **a** the number of OTUs, **b** Shannon index, and **c** Simpson index of the rhizosphere microbial communities between three blueberry varieties and bulk soil samples. Comparison of the similarity of rhizosphere microbial communities between three blueberry varieties and bulk soil samples based on **d** Bray–Curtis and **e** unweighted UniFrac distance metrics

The alpha diversities of rhizosphere microbial communities were compared between three blueberry varieties and bulk soil samples using the number of OTUs, Shannon index, and Simpson index (Fig. 1a–c). We observed that the number of OTUs of microbial communities in bulk soil samples was significantly higher than that in three blueberry varieties, and the number of OTUs of rhizosphere microbial communities among three blueberry varieties was also significantly different (Kruskal**–**Wallis test, *p* < 0.05; Fig. 1a). As for species richness of rhizosphere microbial communities, we found that the Shannon and Simpson indexes of rhizosphere microbial communities of bulk soil samples were significantly higher than those of blueberry varieties, except Southern Highbush Blueberry (Fig. 1b, c).

The similarities of rhizosphere microbial communities were also assessed among three blueberry varieties and bulk soil samples based on Bray**–**Curtis (Fig. 1d) and unweighted UniFrac distance metrics (Fig. 1e). The results of PCoA based on Bray**–**Curtis (Fig. 1d) and unweighted UniFrac distance metrics (Fig. 1e) revealed significant differences in taxonomical compositions between microbial communities of blueberry rhizosphere soil and bulk soil (*p* < 0.001, F = 6.815, One-way PERMANOVA, *N* = 9999, Bray**–**Curtis dissimilarity index). The taxonomical compositions of rhizosphere microbial communities of three blueberry varieties also significantly differed (*p* < 0.001, F = 7.472, One-way PERMANOVA, N = 9999, Bray**–**Curtis dissimilarity index).

### Differential taxonomical composition in blueberry rhizosphere microbial community

**Fig. 2:**
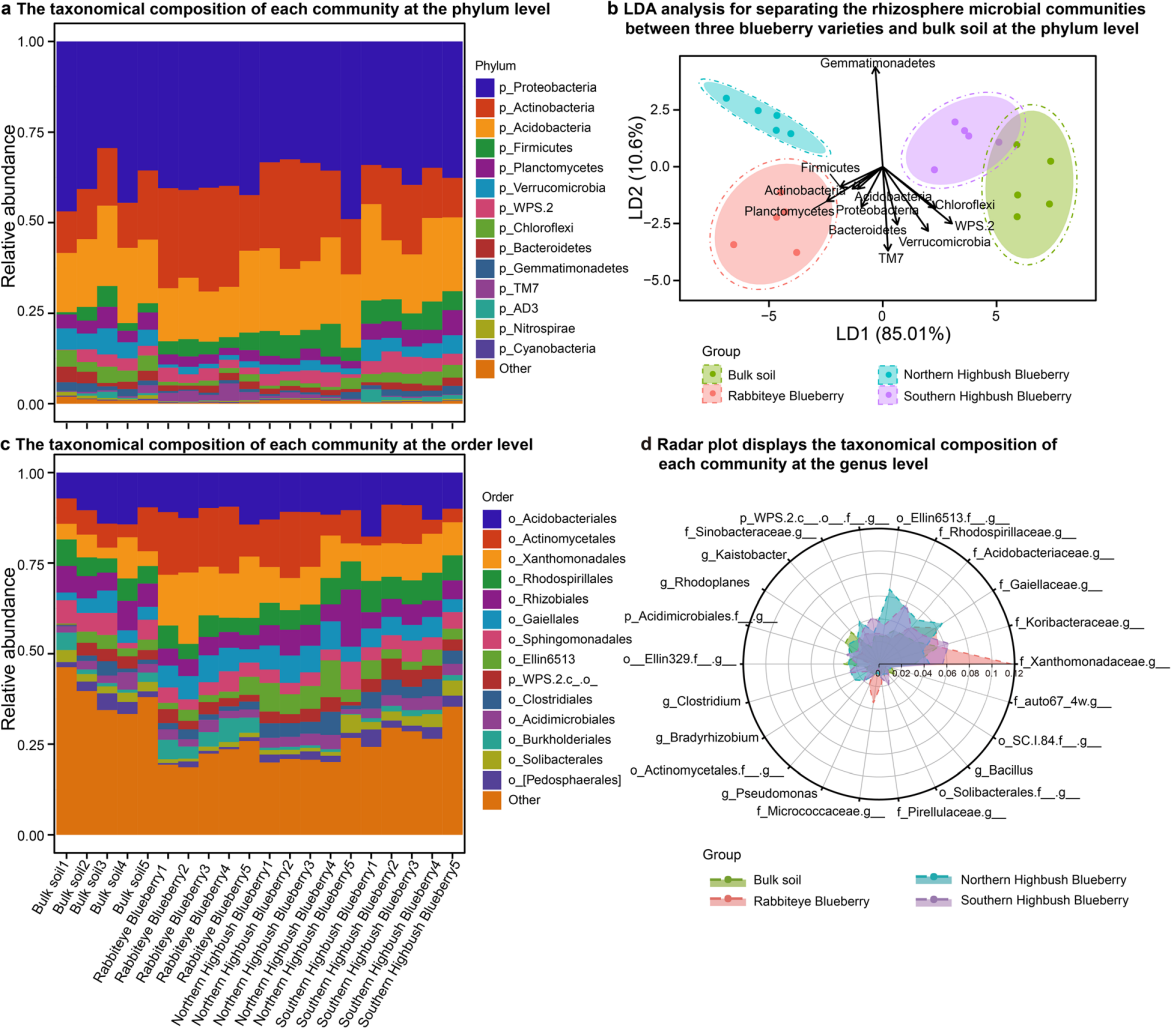
Taxonomical compositions of rhizosphere microbial communities in three blueberry varieties at different taxonomical levels. **a** The taxonomical compositions of rhizosphere microbial community of three blueberry varieties at the phylum level. **b** Linear discriminant analysis was performed to maximize the separation of the rhizosphere microbial communities of three blueberry varieties and bulk soil based on the taxonomical compositions at phylum level. The length and direction of the arrows represent the normalized scaling for each of the predominant phylum. The taxonomical composition of rhizosphere microbial community of three blueberry varieties at **c** the order level. **d** Radar plot displays the average taxonomical composition of rhizosphere microbial community for each blueberry varieties at the genus level

To gain insights into the taxonomical compositions of blueberry rhizosphere microbial communities, we stratified the taxonomical structure of rhizosphere microbial communities at the phylum, order, and genus levels (Fig. 2). We compared the differences in taxonomical compositions between rhizosphere microbial communities of blueberry and bulk soil and among different blueberry varieties.

At the phylum level, we found that *Proteobacteria*, *Actinobacteria*, *Acidobacteria*, *Firmicutes*, *Planctomycetes*, and *Verrucomicrobia* constituted the six most enriched bacterial phyla among rhizosphere microbial community of three blueberry varieties and bulk soil (Fig. 2a). The predominant phylum is almost consistent with a previous study, which also reported that *Proteobacteria*, *Actinobacteria*, *Acidobacteria*, *Bacteroidetes, Planctomycetes*, *Chloroflexi*, and *Verrucomicrobia* were enriched in the rhizosphere microbiome of blueberry [19]. The proportion of *Proteobacteria* of each blueberry variety (Rabbiteye Blueberry: 40.81%±0.87%, Northern Highbush Blueberry: 36.79%±6.2%, Southern Highbush Blueberry: 36.2%±2.07%) was not different from that of bulk soil (39.42%±6.31%, *t*-test, all *p* > 0.05). The relative abundances of *Actinobacteria* of rhizosphere microbial communities of Rabbiteye Blueberry (24.72%±4.91%) and Northern Highbush Blueberry (22.93%±5.49%) varieties were significantly higher than those of bulk soil (14.57%±2.72%, *t*-test, *p* < 0.05). Although the relative abundance of *Firmicutes* increased in rhizosphere microbial communities of three blueberry varieties compared with bulk soil, the proportions in Northern Highbush Blueberry (6.24%±1.8%) and Southern Highbush Blueberry (6.02%±1.13%) were significantly different from that in bulk soil (2.97%±1.72%, *t*-test, *p* < 0.05). The relative abundances of *Nitrospirae* were significantly decreased in rhizosphere microbial communities of Rabbiteye Blueberry (0.26%±0.08%), Northern Highbush Blueberry (0.16%±0.03%), and Southern Highbush Blueberry varieties (0.25%±0.11%) compared with bulk soil (0.7%±0.29%, *t*-test, *p* < 0.05). Additionally, LDA was conducted to maximize the separation of rhizosphere microbial communities of three blueberry varieties and bulk soil based on the relative abundances of predominant phyla. We observed that rhizosphere microbial communities of three blueberry varieties and bulk soil could be distinctly differentiated by integrating a linear combination of phyla (Fig. 2b). Among the linear combination of phyla, we found that *Planctomycetes*, *Gemmatinonadetes*, *Chloroflexi*, and *Verrucomicrobia* were important for differentiating rhizosphere microbial communities of three blueberry varieties and bulk soil (Fig. 2b).

At the order level, we observed that *Acidobacteriales*, *Actinomycetales*, *Xanthomonadales*, *Rhodospirillales*, *Rhizobiales*, and *Gaiellales* were the six predominant bacterial orders in rhizosphere microbial communities of three blueberry varieties and bulk soil (Fig. 2c, Supplementary Figure 1a). Specifically, we found that the average relative abundances of *Actinomycetales* in rhizosphere microbial communities of Rabbiteye Blueberry (15.2%±3.37%) and Northern Highbush Blueberry (12.31%±4.41%) were increased compared with those of bulk soil (7.22%±2.77%) and Southern Highbush Blueberry (6.29%±3.64%). The average relative abundance of *Xanthomonadales* in rhizosphere microbial communities of Rabbiteye Blueberry (15.19%±2.71%) was significantly higher than those of bulk soil (5.81%±2.99%, *t*-test, *p* < 0.01), Northern Highbush Blueberry (9.13%±2.29%, *t*-test, *p* < 0.01), and Southern Highbush Blueberry (9.81%±0.59%, *t*-test, *p* < 0.05). We also profiled the taxonomical composition of rhizosphere microbial communities of blueberry varieties and bulk soil at the genus level, and we found that the specific distribution of genus contributed to the discrepancy of rhizosphere microbial communities (Fig. 2d, Supplementary Figure 1b).

### Differential functional and phenotypic compositions in blueberry rhizosphere microbial community

**Fig. 3:**
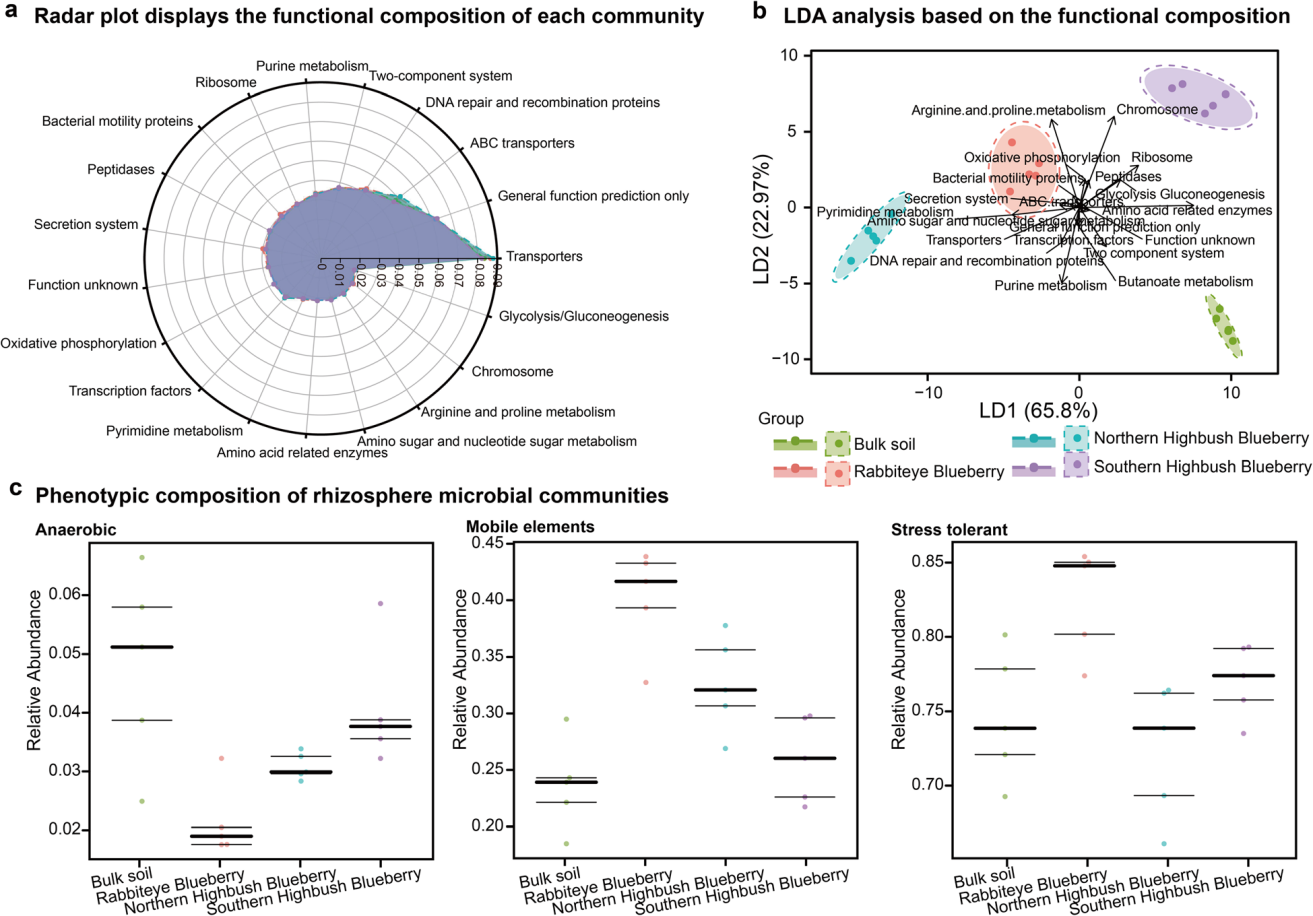
Functional and phenotypic compositions of rhizosphere microbial communities in three blueberry varieties. **a** The functional composition of rhizosphere microbial community of three blueberry varieties. **b** Linear discriminant analysis was performed to distinguish the rhizosphere microbial communities of three blueberry varieties and bulk soil based on the functional composition. **c** Comparison of the phenotypic composition of rhizosphere microbial communities between three blueberry varieties and bulk soil. The phenotype relative abundances were compared using pair-wise Mann–Whitney U tests with false discovery rate correction

The functional and phenotypic compositions in blueberry’s rhizosphere microbial community were profiled based on their taxonomical compositions (Fig. 3). As to the functional traits that collapsed to level 2 of the KEGG database, we found that the enrichment of enzyme families and environmental adaptation in rhizosphere microbial communities and the proportion of biosynthesis of other secondary metabolites was higher in Northern Highbush Blueberry (Supplementary Figure 2). The relative abundances of functional traits related to transporters, general function, ABC transporters, DNA repair and recombination proteins, two-component system, and urine metabolism were higher in the rhizosphere microbial community of blueberry varieties and bulk soil (Fig. 3a). Moreover, we found that the functional compositions of the rhizosphere microbial communities of Rabbiteye Blueberry significantly differed from those of bulk soil (*p* < 0.05, F = 3.545 One–way PERMANOVA, *N* = 9999, Bray–Curtis dissimilarity index) and Southern Highbush Blueberry (*p* < 0.05, F = 3.3, One–way PERMANOVA, *N* = 9999, Bray–Curtis dissimilarity index). The rhizosphere microbial communities of three blueberry varieties and bulk soil could be distinctly distinguished by integrating a linear combination of functional components (Fig. 3b).

Additionally, we explored the phenotypic compositions of rhizosphere microbial communities between three blueberry varieties and bulk soil. We observed that the proportions of anaerobic microbiota, mobile elements, and stress tolerant significantly differed (Kruskal–Wallis test, *p* < 0.05, Fig. 3c). Specifically, the proportions of anaerobic microbiota of bulk soil (4.78%±1.63%) were higher than those of Rabbiteye Blueberry (2.14%±0.62%) and Northern Highbush Blueberry (3.08%±0.23%), except Southern Highbush Blueberry (4.06%±1.04%, Fig. 3c). The relative abundances of mobile elements in the rhizosphere microbial communities of three blueberry varieties (Rabbiteye Blueberry: 40.18%±4.51%, Northern Highbush Blueberry: 32.61%±4.25%, and Southern Highbush Blueberry: 25.95%±3.78%) were higher than those of bulk soil (23.67%±3.99%, Fig. 3c). The proportions of stress tolerant of rhizosphere microbial communities of Rabbiteye Blueberry (82.56%±3.59%) and Southern Highbush Blueberry (77.05%±2.46%), except Northern Highbush Blueberry (72.39%±4.53%), were higher than those of bulk soil (74.64%±4.38%, Fig. 3c).

### Core blueberry rhizosphere microbiome

**Fig. 4:**
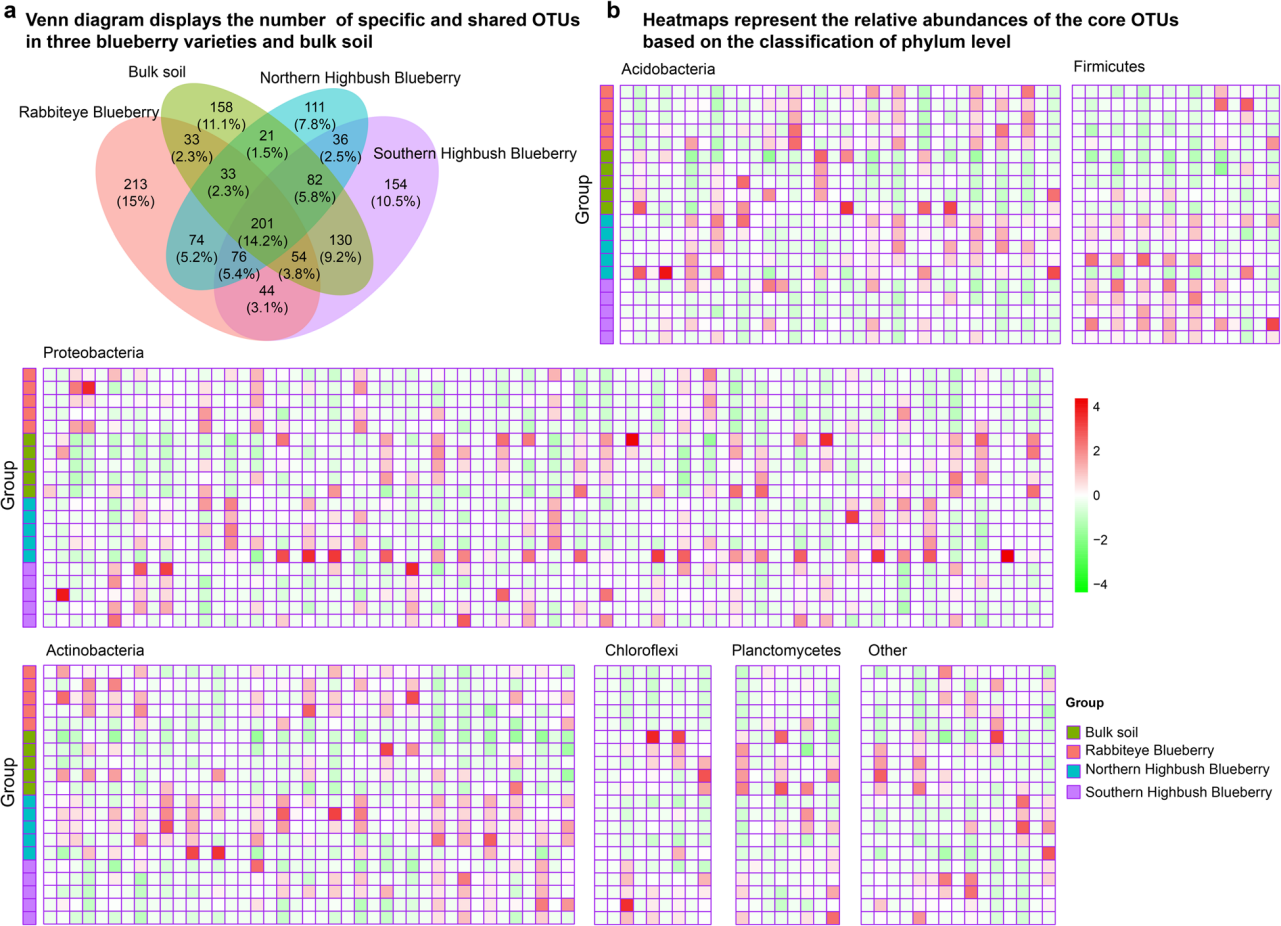
Core taxa in blueberry rhizosphere microbiome. **a** Venn diagram showing specific and shared OTUs across the rhizosphere microbial communities of three blueberry varieties and bulk soil. The shared OTUs were defined as the OTUs appeared in all samples of each group. **b** Heatmaps represent the relative abundances of the core OTUs from all samples of three blueberry varieties and bulk soil. Along the y axis of each heatmap, samples of three blueberry varieties and bulk soil were ordered. The color from green to red represents a relative abundance of each OTU from low to high

We extended our analysis to determine which OTUs are stable across in rhizosphere microbial communities of different blueberry varieties and bulk soil. We identified 728, 634, 777, and 712 OTUs as the core OTUs in rhizosphere microbial communities of Rabbiteye Blueberry, Northern Highbush Blueberry, Southern Highbush Blueberry and bulk soil (Fig. 4a), respectively. Eventually, 201 OTUs of 1420 OTUs (14.2%) were identified as the core OTUs in rhizosphere microbial communities of blueberry varieties and bulk soil (Fig. 4a**,** Supplementary Table 2). Many OTU cases are mainly affiliated with *Proteobacteria* (78 OTUs), *Actinobacteria* (41 OTUs), *Acidobacteria* (34 OTUs), *Firmicutes* (16 OTUs), *Chloroflexi* (9 OTUs), and *Planctomycetes* (8 OTUs, Fig. 4b). The distribution of each core OTU in rhizosphere microbial communities of blueberry varieties was different (Fig. 4b), indicating that the relative abundance of core OTUs varied most among different blueberry varieties.

### Identification of microbial biomarkers for classifying different blueberry varieties

**Fig. 5:**
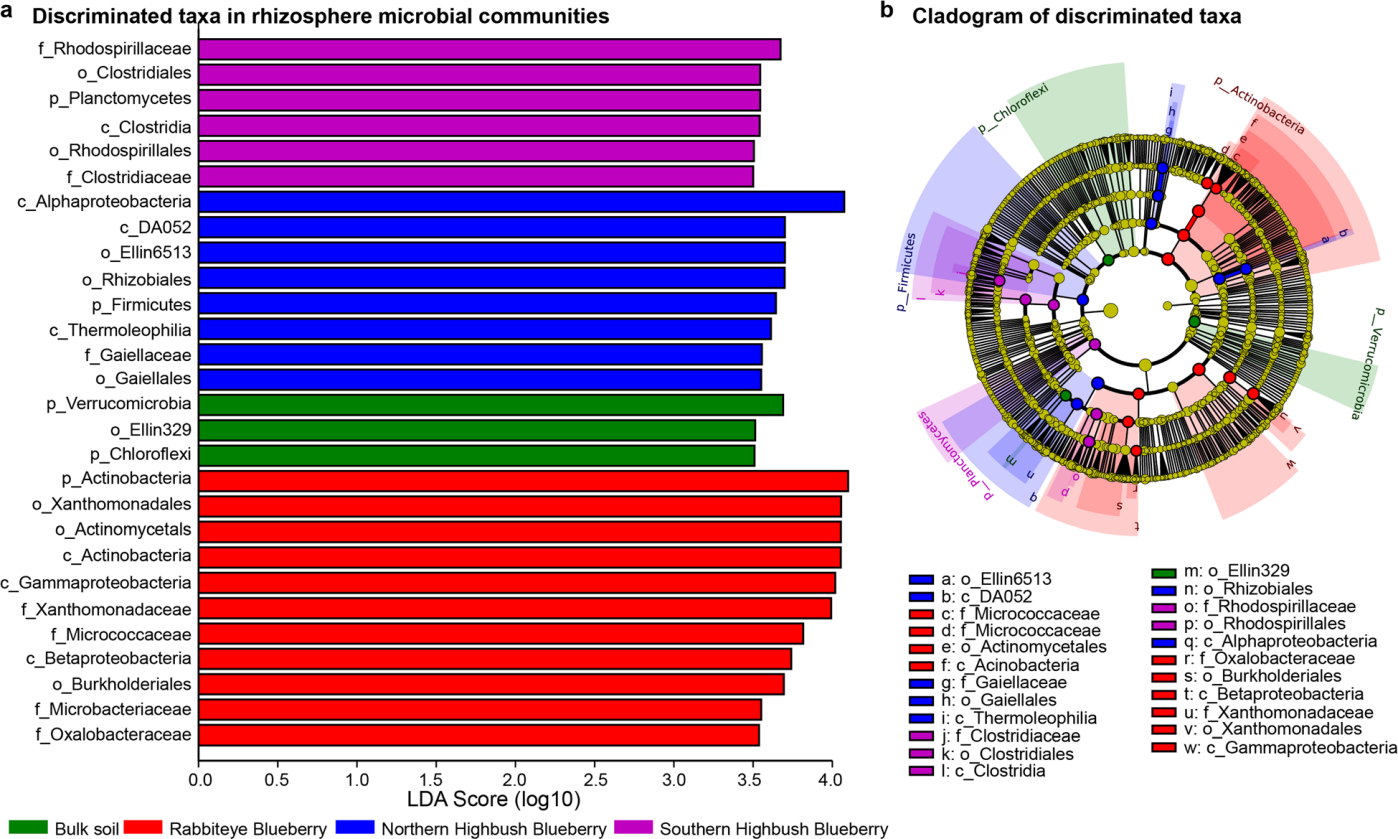
Biomarker analysis of rhizosphere microbial communities of different blueberry varieties and bulk soil. **a** Differentially abundant biomarkers of rhizosphere microbial communities of different blueberry varieties and bulk soil. **b** Cladogram showing the phylogenetic structure of biomarkers for rhizosphere microbial communities of different blueberry varieties and bulk soil

To explore the taxonomical signatures among rhizosphere microbial communities of three blueberry varieties and bulk soil, we conducted LEfSe analysis to identify biomarkers for each blueberry variety based on the taxonomical compositions of rhizosphere microbial communities. Finally, we obtained 28 discriminative biomarkers with logarithmic LDA score > 3.5 (Fig. 5). At the phylum level, we found that *Actinobacteria* and *Planctomycetes* were identified as the biomarkers for Rabbiteye Blueberry and Southern Highbush Blueberry, respectively, whereas *Verrucomincrobia* and *Chloroflexi* were detected as the biomarkers for bulk soil (Fig. 5a). At the order level, we observed *Clostridiales*, *Rhodospirillales*, *Rhizobiales*, *Gaiellales*, *Actinomycetals*, *Xanthomonadales*, and *Burkholderiales* were identified as the biomarkers for three blueberry varieties (Fig. 5).

### Patterns of co-occurrence network in blueberry rhizosphere microbial community

**Fig. 6:**
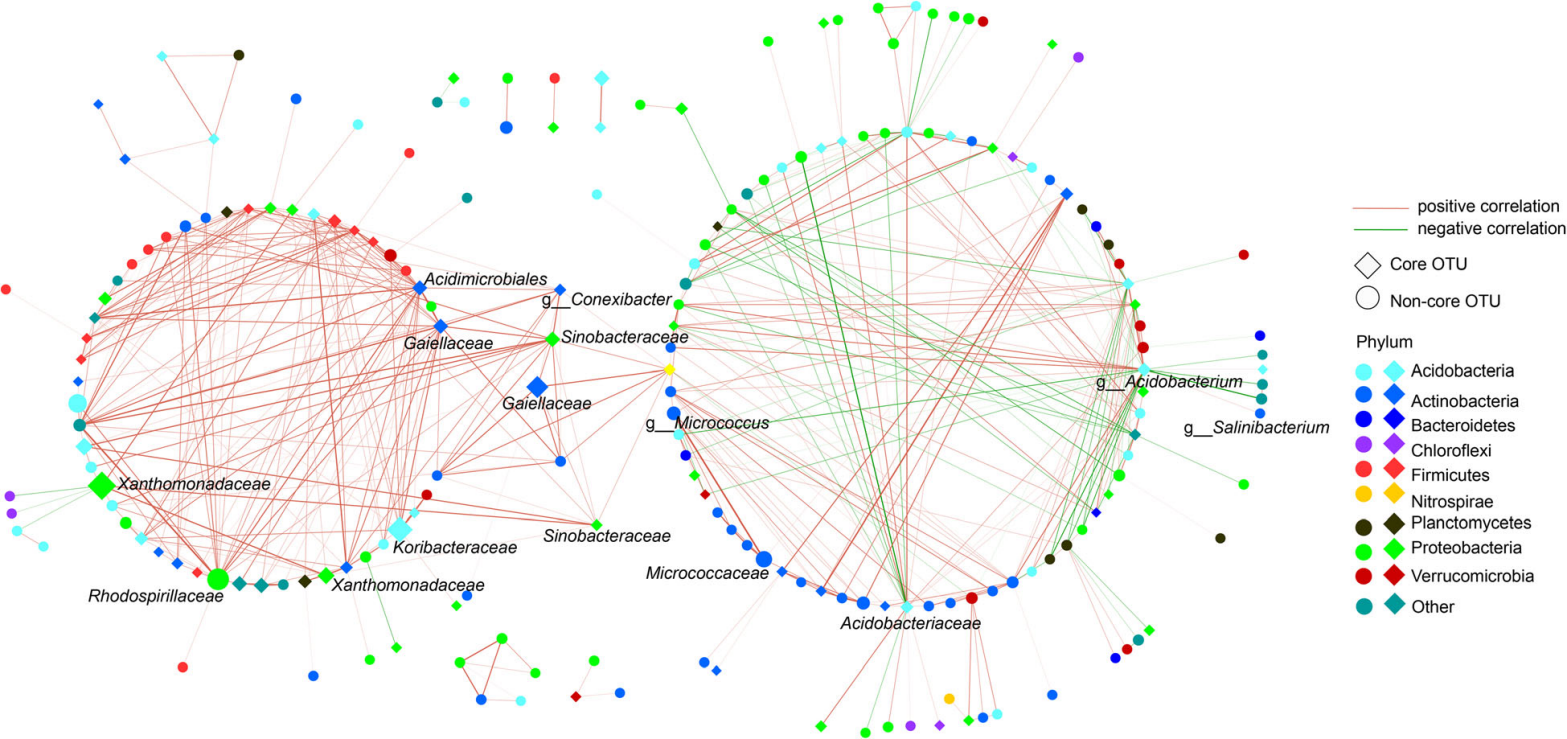
Co-occurrence network of rhizosphere microbial communities of blueberry. The nodes and edges of the co-occurrence network represent the OTUs in the rhizosphere microbial communities of blueberry varieties and the correlations among OTUs, respectively. The colors of edges represent positive and negative correlations among OTUs. The shapes of nodes represent core OTUs and non-core OTUs. The colors of nodes represent the phylum to which OTU belongs

To gain more insights into the interactions among the microbial members of rhizosphere microbial communities of blueberry varieties, we extended our analysis to explore the patterns of OTUs co-occurrence network from an ecological perspective. The SparCC algorithm was applied to calculate the correlations between OTUs and the significant strong correlations (the value of absolute correlations > 0.8 and the *p*-value < 0.05) were chosen to construct the co-occurrence network. The co-occurrence network comprised of 198 nodes and 484 edges (Fig. 6). The density and average degree of the co-occurrence network were 0.025 and 4.89, respectively. The clustering coefficient of the co-occurrence network was 0.35 and the co-occurrence network could be clustered into seven clusters. Strong interactions existed between OTUs in the co-occurrence network. The members of co-occurrence network were mainly affiliated with *Proteobacteria*, *Actinobacteria*, *Acidobacteria*, *Verrucomicrobia*, and *Firmicutes* (Fig. 6). Among the 198 nodes, 74 nodes (37.4%) belonged to core OTUs and these OTUs were mainly affiliated with *Proteobacteria*, *Actinobacteria*, and *Acidobacteria* (Fig. 6). The OTUs with the highest average proportions of the co-occurrence network were members of core OTUs of rhizosphere microbial communities of blueberry varieties, which were affiliated with *Xanthomonadaceae*, *Koribacteraceae*, *Gaiekkaceae*, and *Sinobacteraceae* (Fig. 6).

## Discussion

To obtain a better understanding of the rhizosphere microbiome of blueberry, this pilot study was conducted and mainly focused on the taxonomical, functional, and phenotypic compositions of rhizosphere microbial communities in blueberry. By investigating the compositions of blueberry’s rhizosphere microbial communities and comparing the differences in rhizosphere microbial communities among three blueberry varieties, the characterization of blueberry’s rhizosphere microbial community and the interactions between rhizosphere microbiota should be understood to provide new opportunities to increase the yield of blueberry [3, 15].

Previous studies have reported that plants can shape and recruit protective microorganisms from the soil microbial community to form the rhizosphere microbial community [3, 34], leading to a difference between plants’ rhizosphere microbial community and bulk soil microbial community. In our study, the alpha diversity and beta diversity of rhizosphere microbial communities of blueberry varieties and bulk soil significantly differed. Based on the taxonomical composition, we observed that the microbial diversity of blueberry’s rhizosphere microbial communities decreased compared with bulk soil samples. The decrease in the diversity of rhizosphere microbial communities was also found in a previous study of blueberry focused on the taxonomical composition of bulk soil and rhizosphere microbial communities [18]. Furthermore, the distribution patterns of three blueberry rhizosphere microbial communities and bulk soil were different at the phylum, order, and genus levels. Phyla *Actinobacteria*, *Firmicutes*, and *Planctomycetes* were dominant in the rhizosphere microbial community of three blueberry varieties. In terms of *Firmicutes*, previous studies have reported that the members of *Firmicutes* are identified as groups of bacteria that can confer suppressiveness and important in disease suppressiveness in rhizosphere microbiota of plants [3, 30]. Similarly, *Actinomycetales* was enriched in the blueberry rhizosphere microbial community, which was detected as the dominate group in rhizosphere soil alongside crop growth [35, 36]. The differences in rhizosphere microbial communities between three blueberry varieties and bulk soil samples revealed that a series of microbiota were recruited from the soil microbial community to form the rhizosphere microbial community of blueberry. Additionally, there were significant differences among the rhizosphere microbial communities of three blueberry varieties by comparing the discrepancy of their rhizosphere microbial communities. These results suggested that blueberry can recruit different microbiota to determine the composition of the rhizosphere microbiome and confirmed that different genotype blueberry varieties recruit various microorganisms to form its specific rhizosphere microbiome that contributed to its growth and health [37]. These results were consistent with the differences between plant genotypes even a single gene can contribute a significant impact on the rhizosphere microbiome [38].

Moreover, depth functional profiling analysis revealed that the functional traits were significantly different in rhizosphere microbial communities of blueberry varieties and bulk soil. The increase in functional traits affiliated with enzyme families, environmental adaptation, and biosynthesis of secondary metabolites were associated with the health of blueberry [39, 40]. The phenotypic compositions of different blueberry varieties’ rhizosphere microbial communities also exhibited significant differences. The proportions of stress tolerant of rhizosphere microbial communities of three blueberry varieties were higher than those of bulk soil, which suggested that the rhizosphere microbial composition contributed to different tolerance to stress tolerant for different blueberry varieties [41]. Overall, the differences in functional and phenotypic compositions of microbial communities between rhizosphere microbial communities of three blueberry varieties and bulk soil also suggested that different genotypes of blueberry hold their own unique microbiome, which contributes to their growth and health. The differences in taxonomical, functional, and phenotypic compositions of microbial communities between rhizosphere of blueberry varieties and bulk soil, even among different blueberry varieties, were determined by blueberry genotypes by actively secreting the compounds that specifically stimulate or inhibit the members of the microbial community [42].

Besides, there is core microbiota among the rhizosphere microbial communities of blueberry. We identified 201 OTUs, which were mainly affiliated with *Proteobacteria*, *Actinobacteria*, *Acidobacteria*, *Firmicutes*, *Chloroflexi*, and *Planctomycetes*, as the core rhizosphere microbiota for blueberry rhizosphere microbial communities. Previous studies have reported that beneficial rhizosphere microbiota can directly affect the pathogen in the rhizosphere microbial community [42] and produce the antibiotic compounds and lytic enzymes, consumption of pathogen stimulatory compounds and competitions for nutrients for plants [43]. Among the core microbiota of blueberry, we identified two OTUs affiliated with genus *Pseudomonas* as beneficial rhizosphere microbiota because of rhizosphere *Pseudomonas* spp. can produce the antifungal compound 2,4-diacetylphloroglucinol [44]. Moreover, the rhizosphere microbial compositions of three different blueberry varieties could be distinctly separated, and we selected 28 discriminative biomarkers to distinguish these three blueberry varieties.

Finally, the members of the co-occurrence network and their interactions between OTUs provide a deep understanding of the rhizosphere microbiome of blueberry from an ecological perspective. The members of these families of the rhizosphere microbial community contribute to the growth and health of plants. For example, a previous study reported that the members of *Xanthomonadaceae* family could be divided into non-pathogenic and pathogenic species that infect humans and plants and these species have diverse effects on plant-related lifestyles [45]. The family *Koribacteraceae* of the *Populus trichocarpa* rhizosphere microbiome was reported to be correlated with the production of salicylic acid and populin [46]. Additionally, we observed that *Acidbacterium*, *Salinibacterium*, *Micrococcus*, and *Conexibacter* were involved in co-occurrence network (Fig. 6). Given the limitation of taxonomical classification, the members of these families of rhizosphere microbial communities of blueberry were unclear. Considering the high proportions of these families in co-occurrence network, we need to focus on the functions of these families in future research.

## Conclusions

Our findings highlighted the taxonomical, functional, and phenotypic compositions of the blueberry rhizosphere microbiome and demonstrated the differences of the rhizosphere microbiome in different blueberry varieties. As a result, our study provides an integrative view on the blueberry rhizosphere microbial community and identifies a series of taxa with potential importance from co-occurrence network. The separation of species of core rhizosphere microbiome, especially the beneficial microorganisms, including the non-pathogenic species affiliated with genus *Pseudomonas* and family *Xanthomonadaceae*, could be used as potential microecologics and microbial fertilizers to maintain the health of blueberry during blueberry production. Given that rhizosphere microbiota harbor fungi and bacteria, and mycorrhizosphere interactions can improve plants’ fitness and soil quality [47], the interactions between bacteria and fungi (especially mycorrhizal fungi) should be emphasized in further study. Our present work allows for further investigation into the interactions between bacteria and fungi during blueberry production.

## Methods

### Collection of rhizosphere soil samples

Three blueberry varieties, namely Rabbiteye Blueberry, Northern Highbush Blueberry, and Southern Highbush Blueberry, were selected from a blueberry plantation in Hefei City, Anhui province, China, to investigate the structure of blueberry rhizosphere microbial community and explore the differences among three different blueberry varieties. The selected plants of three blueberry varieties have been planted for 6–7 years. The rhizosphere soil samples of these three blueberry varieties were collected according to the sampling procedure [6, 14] on 13 April 2018 (Supplementary Figure 3). As an artificial plantation, no permission is required for soil collection. Specifically, to obtain the rhizosphere microbiota of blueberry, a small volume of rhizosphere soil was carefully and quickly collected by gently brushing the remaining soil sticking on the blueberry’s roots (the depth of root is about 10 cm) using brush pencils. Five rhizosphere soil samples for each blueberry variety were collected. Five bulk soil samples were also collected at a depth of 10 cm from the surface in the same blueberry plantation where no blueberries and other plants grew and used as control samples. In total, 15 rhizosphere soil samples for three blueberry varieties and five bulk soil samples were collected. These samples were immediately stored in a container at − 20 °C, transported to the laboratory, and stored at − 80 °C.

### DNA extraction and amplicon sequencing

Using PowerSoil DNA Isolation Kit (MoBio, Carlsbad, CA, USA), the total DNA from rhizosphere soil samples of blueberries and bulk soil samples was extracted in Sangon Biotech (Sangon, Shanghai, China), respectively. The concentration and quality of extracted DNAs were quantified using a Qubit® 2.0 Fluorometer (Invitrogen, Carlsbad, CA, USA) and assessed on agarose gels, respectively. The V3–V4 hypervariable regions of the 16S rRNA gene of microbes for each rhizosphere soil sample were amplified and sequenced to profile the structure of the blueberry rhizosphere microbial community. Specifically, approximately 50 ng DNA was used as PCR template, and the forward primer 347F 5′-CCTACGGRRBGCASCAGKVRVGAAT-3′ and reverse primer 802R 5′-GGACTACNVGGGTWTCTAATCC-3′ were used to amplify the V3–V4 amplicons [48]. Indexed adapters were added to the ends of 16S rDNA amplicons and the sequencing library was constructed. The sequencing library was verified, quantified, and sequenced on an Illumina MiSeq platform (San Diego, CA, USA) using the paired-end sequencing strategy in Sangon Biotech (Sangon, Shanghai, China).

### 16S rRNA amplicon data processing and taxonomical profiles

The paired-end reads of 16S rDNA amplicons of each sample were spliced using the Fast Length Adjustment of Short reads (FLASH, v1.2.11) software [49] with default settings. The spliced reads containing ambiguous base calls (N) were removed, and the lengths of spliced reads ranging from 220 bp to 550 bp were chosen by using “trim.seqs” command in the mothur platform [50] (version 1.25.0). The putative chimeras were identified against the SILVA database [51] (release 123) and removed in the mothur platform. The high-quality sequences were used for taxonomical analysis against the Greengenes database [52] (version 13_5) in QIIME (Quantitative Insights Into Microbial Ecology, Boulder, CO, USA, v1.9.1) [53]. The operational taxonomic units (OTUs) were clustered at the 97% nucleotide identity threshold by using the “pick_closed_reference_otus.py” script, and the singletons of OTUs were removed. The final OTU table was rarefied to 18,652 reads per sample prior to downstream analysis to eliminate the effect of sequencing depth.

### Functional and phenotypic compositions of the blueberry rhizosphere microbiome

To compare the differences in functional and phenotypic compositions of rhizosphere microbial communities of different blueberry varieties, two popular tools in current microbiome analysis, namely Phylogenetic Investigation of Communities by Reconstruction of Unobserved States (PICRUSt, version: 1.0.0-dev) [54] and Bugbase [55], were selected to profile the characters of blueberry rhizosphere microbial communities. Specifically, the functional compositions of blueberry rhizosphere microbial communities were predicted The relative abundance of each functional trait that collapsed to levels two and three of the KEGG database (version 66.1, May 1, 2013) was summarized based on the OTU composition. Similarly, the phenotypic compositions of rhizosphere microbial communities, including the content of anaerobic, mobile elements and stress tolerant, were profiled.

### Microbial diversity assessment of blueberry rhizosphere microbial communities

The number of OTUs, Shannon index and Simpson index of rhizosphere soil samples were selected to evaluate the alpha diversities of rhizosphere microbial communities among three blueberry varieties and bulk soil samples. The alpha diversity was compared using the Kruskal–Wallis test among three blueberry varieties and bulk soil. Bray–Curtis distance and unweighted UniFrac metrics (refers to one of the UniFrac metrics, and it only considers the presence or absence of observed microorganisms) [56] were used to compare the differences of beta diversity among three blueberry varieties and bulk soil samples. The clustering result of the rhizosphere microbial community was arrayed by principle coordination analysis (PCoA) and visualized using Emperor [57]. Moreover, linear discriminate analysis (LDA) was performed to utilize a linear combination of features to maximize the separation of rhizosphere microbial communities of three blueberry varieties and bulk soil based on taxonomical composition at the phylum, order, and genus levels. On the basis of the Bray–Curtis distance metric of the taxonomical composition of the genus level, permutational multivariate analysis of variance (PERMANOVA) [58] was used to evaluate whether the rhizosphere microbial communities are significantly different across three blueberry varieties and bulk soil. To determine if other taxa were stable among three blueberry varieties and bulk soil, we identified the core microbiome among rhizosphere samples across groups and visualized the results by venn plot and heatmap in R.

### Biomarker analysis

Linear discriminate analysis effect size (LEfSe, version 1.0) [59] was applied to select the differentially taxonomical features among rhizosphere microbial communities of three blueberry varieties and bulk soil samples. The *p*-value for the factorial Kruskal–Wallis test was set at 0.05 to select statistical significant taxonomical biomarkers. Biomarker with the logarithmic LDA score higher than 3.5 was defined as a discriminative biomarker and visualized.

### Co-occurrence network in blueberry rhizosphere microbial community

The correlations among OTUs of the rhizosphere microbial community of blueberry were calculated using the SparCC algorithm (https://github.com/hallamlab/utilities/wiki/SparCC), which limits the number of spurious correlation identified [60, 61]. The threshold of absolute correlations among OTUs was set at 0.8 and the significant correlations with *p*-value < 0.05 were visualized in Cytoscape [62] (version 3.7.1). The characteristics of the topological structure of the co-occurrence network were analyzed in igraph package [63] (version 0.7.1) in R.

## Supplementary Information

**Additional file 1: Supplementary Figure 1.** Taxonomical composition of rhizosphere microbial communities in three blueberry varieties at the order and genus levels. Linear discriminant analysis was performed to maximize the separation of the rhizosphere microbial communities of three blueberry varieties and bulk soil based on the taxonomical composition at **a:** the order level and **b:** the genus level. The length and direction of the arrows represent the normalized scaling for each predominant phylum. **Supplementary Figure 2.** Functional composition of rhizosphere microbial communities in three blueberry varieties at the level two of the KEGG database. **a**: The functional composition of each rhizosphere microbial community at the level two of the KEGG database. **b**: The average functional traits of each rhizosphere microbial community of the three blueberry varieties and bulk soil. **Supplementary Figure 3.** Sampling schematic for collecting the rhizosphere soil samples of three blueberry varieties and bulk soil samples.

**Additional file 2: Supplementary Table 1.** Number of processed sequencing reads and estimates for the diversity of each microbial community.

**Additional file 3: Supplementary Table 2.** Information of core OTUs in rhizosphere microbial communities of three different blueberry varieties.

## Data Availability

All sequencing data for 15 rhizosphere soil samples of three different blueberry varieties and 5 bulk soil samples were deposited into NCBI’s Sequence Read Archive (SRA) database with the Bioproject number PRJNA574733. The rhizosphere soil samples of Southern Highbush Blueberry, Rabbiteye Blueberry, Northern Highbush Blueberry, and bulk soil samples were labeled with ‘hfon’, ‘hfcn’, ‘hfnf’, and ‘hfcontrol’, respectively.

## Abbreviations

OTUs: Operational taxonomic units
PCoA: Principle coordination analysis
LDA: Linear discriminate analysis
PERMANOVA: Permutational multivariate analysis of variance
LEfSe: Linear discriminate analysis effect size
