# Supplementary material for: Comparison and interpretation of characteristics of Rhizosphere microbiomes of three blueberry varieties
Source: BMC Microbiol. 2021 Jan 22;21:30. doi: 10.1186/s12866-021-02092-7 (PMC7821519; doi:10.1186/s12866-021-02092-7)
Supplement: Supplementary file 1 — Additional file 1: Supplementary Figure 1. Taxonomical composition of rhizosphere microbial communities in three blueberry varieties at the order and genus levels. Linear discriminant analysis was performed to maximize the separation of the rhizosphere microbial communities of three blueberry varieties and bulk soil based on the taxonomical composition at a: the order level and b: the genus level. The length and direction of the arrows represent the normalized scaling for each predominant phylum. Supplementary Figure 2. Functional composition of rhizosphere microbial communities in three blueberry varieties at the level two of the KEGG database. a: The functional composition of each rhizosphere microbial community at the level two of the KEGG database. b: The average functional traits of each rhizosphere microbial community of the three blueberry varieties and bulk soil. Supplementary Figure 3. Sampling schematic for collecting the rhizosphere soil samples of three blueberry varieties and bulk soil samples. [file 12866_2021_2092_MOESM1_ESM.docx]

**Comparison and Interpretation of Characteristics of Rhizosphere Microbiomes of Three Blueberry Varieties**

Yan Zhang^1^, Wei Wang^1^, Zhangjun Shen^1^, Jingjing Wang^1^, Yajun Chen^1^, Dong Wang^1^, Gang Liu^2^, Maozhen Han^2*^

^1^ School of Life Sciences, Hefei Normal University, Hefei, Anhui 230601, China.

^2^ School of Life Sciences, Anhui Medical University, Hefei, Anhui 230032, China.

^*^ Corresponding author E-mail: hanmz@ahmu.edu.cn

**
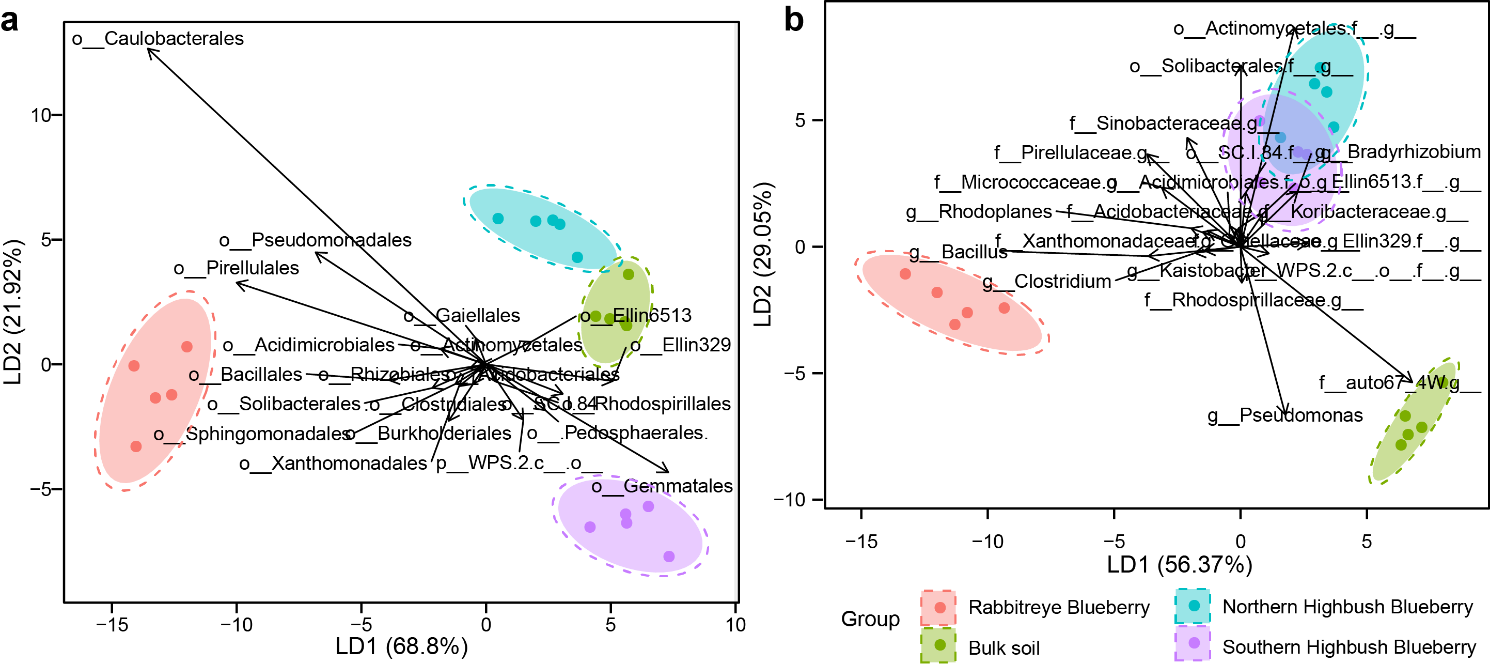
**

**Supplementary Figure 1. Taxonomical composition of rhizosphere microbial communities in three blueberry varieties at the order and genus levels.** Linear discriminant analysis was performed to maximize the separation of the rhizosphere microbial communities of three blueberry varieties and bulk soil based on the taxonomical composition at **a:** the order level and **b:** the genus level. The length and direction of the arrows represent the normalized scaling for each predominant phylum.


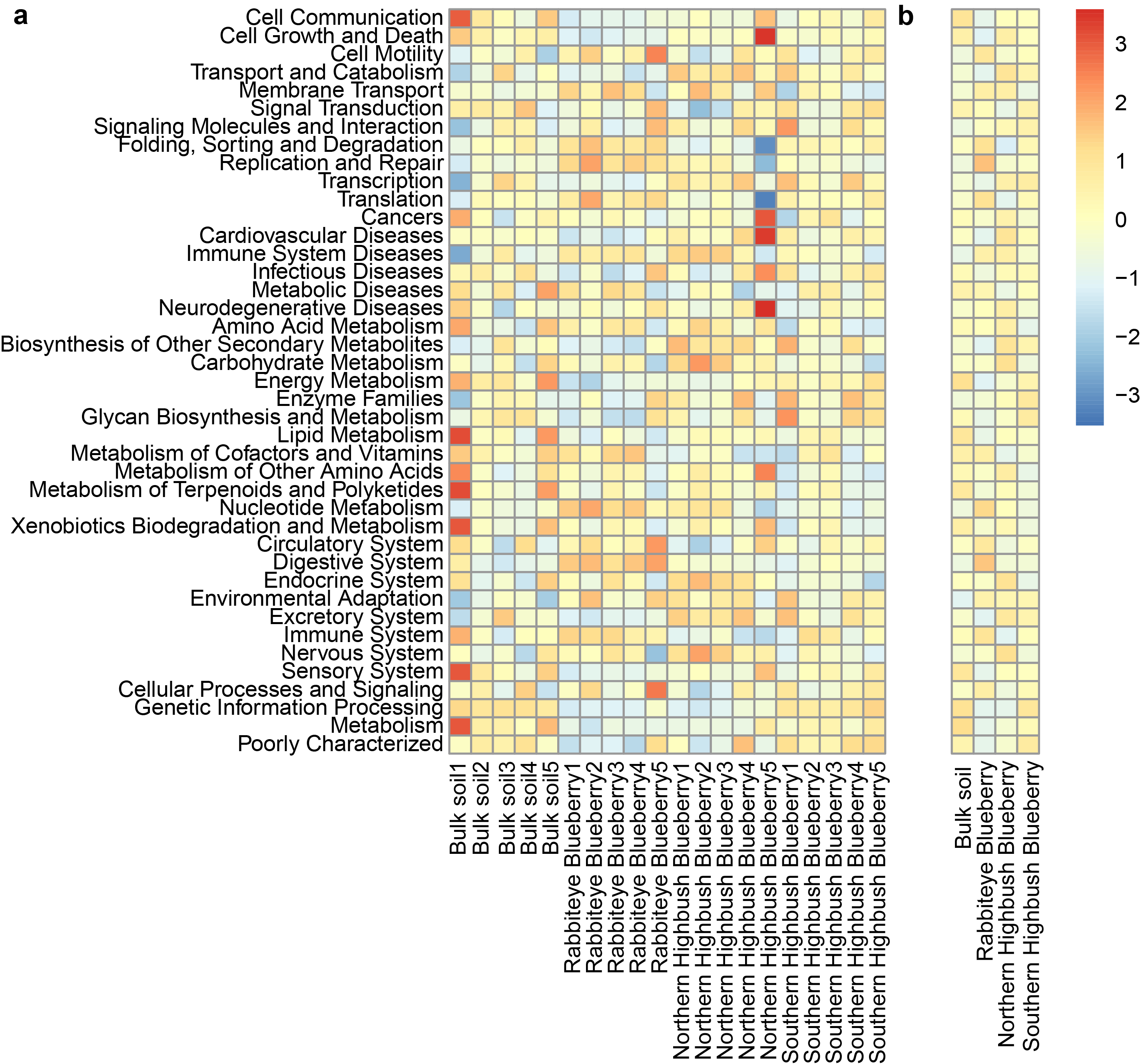


**Supplementary Figure 2. Functional composition of rhizosphere microbial communities in three blueberry varieties at the level 2 of the KEGG database.** **a**: The functional composition of each rhizosphere microbial community at the level 2 of the KEGG database. **b**: The average functional traits of each rhizosphere microbial community of the three blueberry varieties and bulk soil.

**
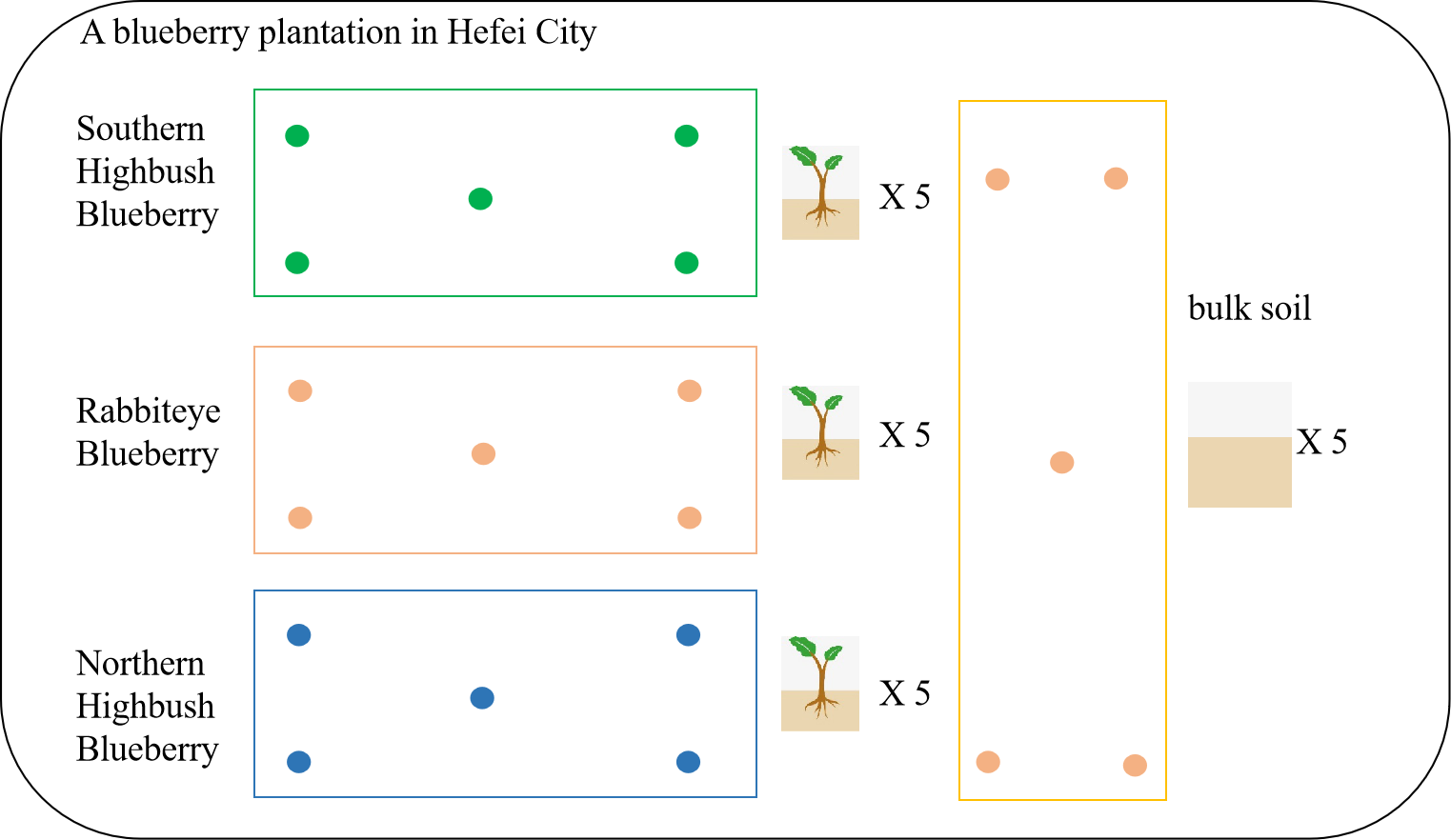
**

**Supplementary Figure 3. Sampling schematic for collecting the rhizosphere soil samples of three blueberry varieties and bulk soil samples.**
