# Supplementary material for: Comparison and interpretation of characteristics of Rhizosphere microbiomes of three blueberry varieties
Source: BMC Microbiol. 2021 Jan 22;21:30. doi: 10.1186/s12866-021-02092-7 (PMC7821519; doi:10.1186/s12866-021-02092-7)
Supplement: Supplementary file 2 — Additional file 2: Supplementary Table 1. Number of processed sequencing reads and estimates for the diversity of each microbial community. [file 12866_2021_2092_MOESM2_ESM.pdf]

Supplementary Table 1. Number of processed sequencing reads and estimates for the diversity of each microbial community

Sample	Raw data	Clean data	Used data	Observed OTUs	Shannon	Chao 1	Simpson
Bulk Soil1	83258	73918	46537	2146	9.230445	3231.839	0.99488515
Bulk Soil2	48961	42517	24432	2421	9.433061	3712.503	0.99601228
Bulk Soil3	51996	46436	25518	2251	9.099705	3429.491	0.99415277
Bulk Soil4	65834	57467	32997	1865	8.709573	3020.399	0.99396142
Bulk Soil5	52241	45042	26035	2548	9.653181	3710.429	0.99656021
Rabbiteye Blueberry1	49258	41775	26941	1845	8.34435	2967.343	0.991005
Rabbiteye Blueberry2	60512	53176	34752	1725	7.911405	2875.88	0.98440264
Rabbiteye Blueberry3	58930	52222	33871	2065	8.622258	3278.245	0.99245111
Rabbiteye Blueberry4	47978	40708	25395	2149	8.685778	3394.933	0.99240688
Rabbiteye Blueberry5	57201	49391	30277	2053	8.375497	3536.385	0.98873309
Northern Highbush Blueberry1	54286	47645	27305	1623	8.202737	2568.156	0.99063691
Northern Highbush Blueberry2	48791	41985	24396	1597	8.41084	2500.973	0.99278961
Northern Highbush Blueberry3	70267	62022	35419	1654	8.346727	2611.737	0.99174458
Northern Highbush Blueberry4	64119	56717	30378	1557	7.974618	2432.548	0.98789479
Northern Highbush Blueberry5	62380	53645	32754	1572	8.502944	2357.987	0.99279721
Southern Highbush Blueberry1	76321	70488	45986	1495	7.819656	2450.277	0.9872672
Southern Highbush Blueberry2	44543	37328	22698	2221	9.152915	3356.637	0.99525945
Southern Highbush Blueberry3	39317	31591	18652	2248	9.251683	3453.128	0.99585147
Southern Highbush Blueberry4	53433	45417	27334	1836	8.492031	2788.503	0.99191247
Southern Highbush Blueberry5	56481	48223	28578	2199	9.230829	3142.623	0.99580421

Note: As to the values of Shannon, Chao 1 and PD whole tree were the maximum value obtained from the results of QIIME.
