# Supplementary material for: Comparison and interpretation of characteristics of Rhizosphere microbiomes of three blueberry varieties
Source: BMC Microbiol. 2021 Jan 22;21:30. doi: 10.1186/s12866-021-02092-7 (PMC7821519; doi:10.1186/s12866-021-02092-7)
Supplement: Supplementary file 3 — Additional file 3: Supplementary Table 2. Information of core OTUs in rhizosphere microbial communities of three different blueberry varieties. [file 12866_2021_2092_MOESM3_ESM.pdf]

Supplementary Table 2. Information of core OTUs in rhizosphere microbial communities of three different blueberry varieties.

OTU ID	Rabbiteye Blueberry 1	Rabbiteye Blueberry 2	Rabbiteye Blueberry 3	Rabbiteye Blueberry 4	Rabbiteye Blueberry 5	Bulk soil1	Bulk soil2	Bulk soil 3	Bulk soil4	Bulk soil5	Northern Highbush Blueberry 1	Northern Highbush Blueberry 2	Northern Highbush Blueberry 3	Northern Highbush Blueberry 4	Northern Highbush Blueberry 5	Southern Highbush Blueberry 1	Southern Highbush Blueberry 2	Southern Highbush Blueberry 3	Southern Highbush Blueberry4	Southern Highbush Blueberry 5	taxonomy
728640	1	1	4	4	4	6	12	67	27	9	18	37	17	18	80	2	9	6	3	6	k__Bacteria; p__Acidobacteria; c__Acidobacteria-6; o__iii1-15; f__; g__; s__
759747	6	3	3	7	20	72	21	4	5	18	10	6	10	5	11	3	11	16	8	13	k__Bacteria; p__Acidobacteria; c__Acidobacteria-6; o__iii1-15; f__; g__; s__
846759	16	9	14	13	8	2	2	1	2	1	7	5	11	8	3	1	1	5	2	3	k__Bacteria; p__Acidobacteria; c__Acidobacteriia; o__Acidobacteriales; f__Acidobacteriaceae; g__; s__
1138433	9	6	6	7	5	3	3	4	8	6	2	9	4	2	1	1	2	2	2	2	k__Bacteria; p__Acidobacteria; c__Acidobacteriia; o__Acidobacteriales; f__Acidobacteriaceae; g__; s__
156636	16	25	8	38	27	6	3	2	1	3	21	27	32	13	16	2	2	4	4	7	k__Bacteria; p__Acidobacteria; c__Acidobacteriia; o__Acidobacteriales; f__Acidobacteriaceae; g__; s__
553387	1	3	2	1	1	11	9	6	5	10	9	6	11	9	11	18	6	12	13	11	k__Bacteria; p__Acidobacteria; c__Acidobacteriia; o__Acidobacteriales; f__Acidobacteriaceae; g__; s__
41797	18	17	14	15	18	2	6	5	8	6	16	18	17	15	7	4	6	11	9	14	k__Bacteria; p__Acidobacteria; c__Acidobacteriia; o__Acidobacteriales; f__Acidobacteriaceae; g__; s__
3990161	2	3	3	5	19	2	17	18	18	14	69	44	59	47	57	105	66	85	122	82	k__Bacteria; p__Acidobacteria; c__Acidobacteriia; o__Acidobacteriales; f__Acidobacteriaceae; g__; s__
237338	10	7	5	10	6	3	10	5	29	8	17	9	18	14	8	5	15	9	7	6	k__Bacteria; p__Acidobacteria; c__Acidobacteriia; o__Acidobacteriales; f__Acidobacteriaceae; g__; s__
699085	163	72	156	69	180	3	44	85	97	24	74	59	74	143	19	63	110	64	108	76	k__Bacteria; p__Acidobacteria; c__Acidobacteriia; o__Acidobacteriales; f__Acidobacteriaceae; g__; s__
239787	11	12	14	8	13	176	82	71	280	128	67	111	87	76	226	136	86	92	135	122	k__Bacteria; p__Acidobacteria; c__Acidobacteriia; o__Acidobacteriales; f__Acidobacteriaceae; g__; s__
4401025	20	63	18	18	35	21	23	17	21	14	24	29	15	9	29	31	28	53	43	100	k__Bacteria; p__Acidobacteria; c__Acidobacteriia; o__Acidobacteriales; f__Acidobacteriaceae; g__; s__
512006	139	122	124	97	162	25	25	26	30	17	155	133	172	107	65	24	15	27	30	20	k__Bacteria; p__Acidobacteria; c__Acidobacteriia; o__Acidobacteriales; f__Acidobacteriaceae; g__; s__
877844	58	27	51	28	41	39	53	21	240	47	148	100	127	126	91	58	36	52	56	25	k__Bacteria; p__Acidobacteria; c__Acidobacteriia; o__Acidobacteriales; f__Acidobacteriaceae; g__; s__
218730	266	124	217	146	192	87	131	146	173	105	235	189	198	194	205	255	156	153	248	200	k__Bacteria; p__Acidobacteria; c__Acidobacteriia; o__Acidobacteriales; f__Acidobacteriaceae; g__; s__
1003326	193	314	211	158	176	53	28	19	36	30	194	215	211	157	109	11	24	20	13	13	k__Bacteria; p__Acidobacteria; c__Acidobacteriia; o__Acidobacteriales; f__Acidobacteriaceae; g__Acidobacterium; s__
255143	39	23	32	13	38	76	25	24	111	24	21	22	18	37	4	7	24	14	23	16	k__Bacteria; p__Acidobacteria; c__Acidobacteriia; o__Acidobacteriales; f__Acidobacteriaceae; g__Telmatobacter; s__bradus
284815	29	44	18	19	51	1	42	93	30	25	32	22	17	39	28	63	41	37	92	35	k__Bacteria; p__Acidobacteria; c__Acidobacteriia; o__Acidobacteriales; f__Koribacteraceae; g__; s__
109694	5	5	6	14	5	49	34	33	16	34	15	7	1	6	27	2	4	8	3	13	k__Bacteria; p__Acidobacteria; c__Acidobacteriia; o__Acidobacteriales; f__Koribacteraceae; g__; s__
744237	10	106	14	15	135	4	53	96	58	45	21	17	13	23	41	193	58	40	66	67	k__Bacteria; p__Acidobacteria; c__Acidobacteriia; o__Acidobacteriales; f__Koribacteraceae; g__; s__
546509	77	87	110	159	164	16	28	26	45	20	26	35	28	33	28	17	9	9	19	10	k__Bacteria; p__Acidobacteria; c__Acidobacteriia; o__Acidobacteriales; f__Koribacteraceae; g__; s__
791077	21	349	22	18	373	36	129	237	167	100	80	38	50	49	126	838	131	134	243	152	k__Bacteria; p__Acidobacteria; c__Acidobacteriia; o__Acidobacteriales; f__Koribacteraceae; g__; s__
636026	464	539	363	528	591	27	407	844	429	320	461	308	323	482	154	906	411	323	685	433	k__Bacteria; p__Acidobacteria; c__Acidobacteriia; o__Acidobacteriales; f__Koribacteraceae; g__; s__
665808	125	104	89	51	62	32	97	87	181	61	174	161	139	197	65	239	59	94	141	68	k__Bacteria; p__Acidobacteria; c__Acidobacteriia; o__Acidobacteriales; f__Koribacteraceae; g__; s__
638638	33	11	22	55	6	21	156	44	122	33	149	51	17	25	15	2	4	4	1	3	k__Bacteria; p__Acidobacteria; c__Acidobacteriia; o__Acidobacteriales; f__Koribacteraceae; g__Candidatus Koribacter; s__
4354098	5	2	12	6	1	100	65	113	44	52	42	24	16	29	100	34	29	37	39	38	k__Bacteria; p__Acidobacteria; c__Acidobacteriia; o__Acidobacteriales; f__Koribacteraceae; g__Candidatus Koribacter; s__
577299	2	1	3	5	1	1	11	12	14	11	23	17	15	9	22	7	7	2	8	13	k__Bacteria; p__Acidobacteria; c__DA052; o__Ellin6513; f__; g__; s__
559586	86	198	79	76	216	5	50	58	139	43	153	130	97	144	41	239	114	116	212	112	k__Bacteria; p__Acidobacteria; c__DA052; o__Ellin6513; f__; g__; s__
584055	165	233	196	174	420	6	83	152	36	75	382	244	306	425	415	358	158	139	311	167	k__Bacteria; p__Acidobacteria; c__DA052; o__Ellin6513; f__; g__; s__
729167	8	2	3	8	10	11	4	6	1	8	20	9	11	23	19	8	13	16	5	11	k__Bacteria; p__Acidobacteria; c__Solibacteres; o__Solibacterales; f__; g__; s__
693971	2	3	3	10	2	14	4	5	1	8	3	7	9	6	47	3	8	9	12	14	k__Bacteria; p__Acidobacteria; c__Solibacteres; o__Solibacterales; f__; g__; s__
627700	61	27	63	52	31	1	8	7	2	5	39	45	42	51	207	7	10	5	27	4	k__Bacteria; p__Acidobacteria; c__Solibacteres; o__Solibacterales; f__Solibacteraceae; g__; s__
204875	2	2	3	1	1	40	33	45	97	29	26	12	22	15	99	11	15	19	18	12	k__Bacteria; p__Acidobacteria; c__Solibacteres; o__Solibacterales; f__Solibacteraceae; g__Candidatus Solibacter; s__
1110404	11	15	14	9	25	4	23	20	42	15	56	26	47	64	29	106	69	58	163	136	k__Bacteria; p__Acidobacteria; c__TM1; o__; f__; g__; s__
211094	4	6	10	7	11	2	6	1	5	1	10	6	12	11	6	7	7	6	13	5	k__Bacteria; p__Actinobacteria; c__Acidimicrobiia; o__Acidimicrobiales; f__; g__; s__
2625114	155	128	204	93	146	1	40	40	46	30	73	113	75	88	13	61	80	75	144	91	k__Bacteria; p__Actinobacteria; c__Acidimicrobiia; o__Acidimicrobiales; f__; g__; s__
161090	173	137	153	92	113	5	103	122	74	80	137	138	181	217	37	247	231	243	328	212	k__Bacteria; p__Actinobacteria; c__Acidimicrobiia; o__Acidimicrobiales; f__; g__; s__
899404	12	3	7	6	10	1	5	10	10	10	13	5	11	22	8	14	5	5	20	12	k__Bacteria; p__Actinobacteria; c__Actinobacteria; o__Actinomycetales; f__; g__; s__
937735	12	6	18	11	3	1	15	5	23	11	15	16	12	13	10	13	7	6	11	5	k__Bacteria; p__Actinobacteria; c__Actinobacteria; o__Actinomycetales; f__; g__; s__
1043812	2	4	5	1	1	1	9	14	3	5	12	8	26	20	2	17	51	42	43	28	k__Bacteria; p__Actinobacteria; c__Actinobacteria; o__Actinomycetales; f__; g__; s__
814609	15	15	13	2	12	2	12	13	18	7	31	26	24	46	30	13	21	21	22	21	k__Bacteria; p__Actinobacteria; c__Actinobacteria; o__Actinomycetales; f__; g__; s__
210410	6	6	15	11	7	3	14	30	33	15	52	27	79	68	5	43	127	85	106	68	k__Bacteria; p__Actinobacteria; c__Actinobacteria; o__Actinomycetales; f__; g__; s__
334140	6	12	10	9	8	3	10	32	7	16	38	34	37	43	20	20	59	40	30	40	k__Bacteria; p__Actinobacteria; c__Actinobacteria; o__Actinomycetales; f__; g__; s__
566039	19	14	9	7	4	6	6	21	3	9	13	17	20	12	9	9	11	15	14	8	k__Bacteria; p__Actinobacteria; c__Actinobacteria; o__Actinomycetales; f__; g__; s__
668615	34	20	36	22	15	9	56	104	29	30	117	89	111	160	40	84	96	78	100	83	k__Bacteria; p__Actinobacteria; c__Actinobacteria; o__Actinomycetales; f__; g__; s__
865748	39	18	42	14	16	6	15	25	14	27	25	25	34	17	12	4	12	10	11	12	k__Bacteria; p__Actinobacteria; c__Actinobacteria; o__Actinomycetales; f__Frankiaceae; g__; s__
259094	18	25	44	19	13	15	16	13	13	33	19	18	20	16	10	5	10	13	11	13	k__Bacteria; p__Actinobacteria; c__Actinobacteria; o__Actinomycetales; f__Frankiaceae; g__Kaistibacter; s__ginsenosidimutans
939546	150	81	152	65	23	3	23	27	12	30	72	44	43	44	23	20	33	22	19	11	k__Bacteria; p__Actinobacteria; c__Actinobacteria; o__Actinomycetales; f__Geodermatophilaceae; g__Geodermatophilus; s__
885496	54	31	57	68	20	21	56	49	13	137	22	36	34	7	8	3	29	77	2	5	k__Bacteria; p__Actinobacteria; c__Actinobacteria; o__Actinomycetales; f__Intrasporangiaceae; g__; s__
1074625	25	66	20	27	5	1	12	4	3	14	16	23	11	3	13	2	15	19	1	1	k__Bacteria; p__Actinobacteria; c__Actinobacteria; o__Actinomycetales; f__Microbacteriaceae; g__; s__
1108350	47	76	47	34	26	2	35	29	13	35	86	106	84	11	61	4	68	59	4	12	k__Bacteria; p__Actinobacteria; c__Actinobacteria; o__Actinomycetales; f__Microbacteriaceae; g__Microbacterium; s__
959160	24	58	19	36	7	1	4	2	2	6	27	37	29	9	17	1	17	18	2	3	k__Bacteria; p__Actinobacteria; c__Actinobacteria; o__Actinomycetales; f__Microbacteriaceae; g__Mycetocola; s__
223583	19	5	16	15	9	3	3	2	1	2	6	35	14	7	4	7	15	16	8	4	k__Bacteria; p__Actinobacteria; c__Actinobacteria; o__Actinomycetales; f__Mycobacteriaceae; g__Mycobacterium; s__celatum
1033426	4	11	39	16	7	25	38	55	14	97	9	21	18	6	30	5	20	29	4	18	k__Bacteria; p__Actinobacteria; c__Actinobacteria; o__Actinomycetales; f__Nocardioidaceae; g__Nocardioides; s__
869089	18	26	25	38	11	9	20	23	7	15	22	17	16	19	14	14	11	24	16	16	k__Bacteria; p__Actinobacteria; c__Actinobacteria; o__Actinomycetales; f__Sporichthyaceae; g__; s__
1087325	27	55	24	70	91	3	19	16	8	19	96	171	112	17	53	2	26	53	1	3	k__Bacteria; p__Actinobacteria; c__Actinobacteria; o__Actinomycetales; f__Streptomycetaceae; g__; s__
994098	63	99	63	90	194	4	42	61	17	42	225	344	239	41	55	3	144	160	3	7	k__Bacteria; p__Actinobacteria; c__Actinobacteria; o__Actinomycetales; f__Streptomycetaceae; g__; s__
4444121	10	4	13	3	7	1	1	4	1	2	14	6	15	25	2	11	30	12	39	14	k__Bacteria; p__Actinobacteria; c__Thermoleophilia; o__Gaiellales; f__Gaiellaceae; g__; s__
208423	178	141	200	183	104	1	109	106	78	57	185	184	209	267	39	347	154	132	178	113	k__Bacteria; p__Actinobacteria; c__Thermoleophilia; o__Gaiellales; f__Gaiellaceae; g__; s__
210607	18	19	25	35	3	3	9	9	1	11	7	7	11	5	4	1	10	5	3	3	k__Bacteria; p__Actinobacteria; c__Thermoleophilia; o__Gaiellales; f__Gaiellaceae; g__; s__
141167	9	15	23	17	1	2	15	14	7	6	20	12	6	8	3	9	7	6	7	4	k__Bacteria; p__Actinobacteria; c__Thermoleophilia; o__Gaiellales; f__Gaiellaceae; g__; s__
368831	1	4	2	3	3	8	8	31	7	11	16	22	9	3	51	4	7	13	10	3	k__Bacteria; p__Actinobacteria; c__Thermoleophilia; o__Gaiellales; f__Gaiellaceae; g__; s__
4327554	13	12	22	18	2	5	10	20	8	11	11	20	16	14	10	9	14	23	13	8	k__Bacteria; p__Actinobacteria; c__Thermoleophilia; o__Gaiellales; f__Gaiellaceae; g__; s__
568461	6	16	12	8	6	3	4	15	14	16	35	41	40	33	72	8	12	6	13	7	k__Bacteria; p__Actinobacteria; c__Thermoleophilia; o__Gaiellales; f__Gaiellaceae; g__; s__
539978	14	6	9	17	4	13	3	12	11	7	5	12	4	4	11	3	6	3	7	3	k__Bacteria; p__Actinobacteria; c__Thermoleophilia; o__Gaiellales; f__Gaiellaceae; g__; s__
566165	6	23	16	22	5	3	13	20	44	8	57	65	82	35	17	8	9	9	16	12	k__Bacteria; p__Actinobacteria; c__Thermoleophilia; o__Gaiellales; f__Gaiellaceae; g__; s__
240388	16	20	19	33	16	3	23	36	11	10	21	23	31	28	10	20	14	23	22	17	k__Bacteria; p__Actinobacteria; c__Thermoleophilia; o__Gaiellales; f__Gaiellaceae; g__; s__
368027	20	11	21	26	17	6	10	7	6	7	15	19	17	25	13	15	3	5	8	4	k__Bacteria; p__Actinobacteria; c__Thermoleophilia; o__Gaiellales; f__Gaiellaceae; g__; s__
1037256	16	47	23	17	16	16	12	34	19	15	7	6	8	7	5	6	23	30	20	16	k__Bacteria; p__Actinobacteria; c__Thermoleophilia; o__Gaiellales; f__Gaiellaceae; g__; s__
755123	36	74	48	50	22	8	35	66	24	45	25	56	46	28	45	9	41	33	35	23	k__Bacteria; p__Actinobacteria; c__Thermoleophilia; o__Gaiellales; f__Gaiellaceae; g__; s__
220013	55	37	53	58	21	14	24	66	20	35	56	62	57	70	36	26	79	70	77	52	k__Bacteria; p__Actinobacteria; c__Thermoleophilia; o__Gaiellales; f__Gaiellaceae; g__; s__
214128	82	149	129	136	57	66	57	138	50	102	53	84	67	47	114	37	35	30	31	41	k__Bacteria; p__Actinobacteria; c__Thermoleophilia; o__Gaiellales; f__Gaiellaceae; g__; s__
936318	415	360	530	348	277	83	217	296	226	137	569	597	561	672	233	353	279	239	406	192	k__Bacteria; p__Actinobacteria; c__Thermoleophilia; o__Gaiellales; f__Gaiellaceae; g__; s__
217392	35	11	46	15	10	1	7	36	17	11	19	25	23	22	3	18	15	10	20	16	k__Bacteria; p__Actinobacteria; c__Thermoleophilia; o__Solirubrobacterales; f__Conexibacteraceae; g__; s__
574032	133	55	148	58	45	1	41	33	31	25	119	138	119	78	37	50	166	97	70	47	k__Bacteria; p__Actinobacteria; c__Thermoleophilia; o__Solirubrobacterales; f__Conexibacteraceae; g__Conexibacter; s__
212027	3	2	3	3	4	8	17	29	19	7	2	3	4	3	8	1	17	10	8	7	k__Bacteria; p__Chloroflexi; c__Ellin6529; o__; f__; g__; s__
112867	4	4	9	13	7	21	30	23	5	51	4	14	8	1	20	4	18	33	8	14	k__Bacteria; p__Chloroflexi; c__Ellin6529; o__; f__; g__; s__
849821	5	3	4	5	4	21	11	3	4	12	1	7	3	1	12	1	6	7	7	8	k__Bacteria; p__Chloroflexi; c__Ellin6529; o__; f__; g__; s__
775409	3	1	2	6	6	17	21	45	23	17	2	4	5	3	15	6	20	14	11	17	k__Bacteria; p__Chloroflexi; c__Ellin6529; o__; f__; g__; s__
284171	2	1	1	4	4	50	17	11	10	18	1	7	9	1	7	5	9	15	13	17	k__Bacteria; p__Chloroflexi; c__Ellin6529; o__; f__; g__; s__
128177	20	35	45	44	67	65	52	118	34	65	23	49	21	19	109	30	37	39	26	56	k__Bacteria; p__Chloroflexi; c__Ellin6529; o__; f__; g__; s__
822323	3	3	3	1	4	13	6	21	24	28	5	4	7	7	7	40	46	31	108	47	k__Bacteria; p__Chloroflexi; c__Ktedonobacteria; o__JG30-KF-AS9; f__; g__; s__
204816	5	8	17	1	11	38	8	9	21	17	8	21	15	10	43	68	31	16	82	23	k__Bacteria; p__Chloroflexi; c__Ktedonobacteria; o__JG30-KF-AS9; f__; g__; s__
4300477	9	8	17	4	19	50	18	10	63	23	23	32	21	21	82	31	28	30	46	28	k__Bacteria; p__Chloroflexi; c__Ktedonobacteria; o__JG30-KF-AS9; f__; g__; s__
823024	4	5	4	2	30	2	19	8	2	2	25	2	4	9	1	3	9	10	45	14	k__Bacteria; p__Firmicutes; c__Bacilli; o__Bacillales; f__Bacillaceae; g__Bacillus; s__
697578	4	5	10	3	10	8	2	8	7	1	4	7	7	3	26	13	6	1	3	6	k__Bacteria; p__Firmicutes; c__Bacilli; o__Bacillales; f__Bacillaceae; g__Bacillus; s__
544851	5	13	7	3	3	3	3	2	3	7	8	1	5	5	11	5	4	3	2	2	k__Bacteria; p__Firmicutes; c__Bacilli; o__Bacillales; f__Bacillaceae; g__Bacillus; s__
1051517	110	114	122	93	202	12	80	88	50	40	173	83	74	149	31	165	103	79	275	126	k__Bacteria; p__Firmicutes; c__Bacilli; o__Bacillales; f__Bacillaceae; g__Bacillus; s__
695185	25	35	16	16	21	5	15	14	17	2	19	15	11	18	12	23	12	15	28	15	k__Bacteria; p__Firmicutes; c__Bacilli; o__Bacillales; f__Bacillaceae; g__Bacillus; s__foraminis
563918	12	27	11	14	19	2	18	38	16	12	21	23	29	56	6	68	55	37	70	49	k__Bacteria; p__Firmicutes; c__Clostridia; o__Clostridiales; f__Clostridiaceae; g__; s__
837039	2	4	4	4	2	1	3	6	2	3	11	10	6	13	1	11	15	10	10	9	k__Bacteria; p__Firmicutes; c__Clostridia; o__Clostridiales; f__Clostridiaceae; g__Clostridium; s__
555562	5	6	6	2	4	1	3	19	2	2	15	15	17	25	5	5	14	6	6	9	k__Bacteria; p__Firmicutes; c__Clostridia; o__Clostridiales; f__Clostridiaceae; g__Clostridium; s__
593302	17	25	13	11	25	1	14	40	7	6	38	34	20	55	8	65	38	38	51	46	k__Bacteria; p__Firmicutes; c__Clostridia; o__Clostridiales; f__Clostridiaceae; g__Clostridium; s__
541432	5	4	7	2	7	1	2	14	2	4	9	14	10	24	3	4	10	7	13	10	k__Bacteria; p__Firmicutes; c__Clostridia; o__Clostridiales; f__Clostridiaceae; g__Clostridium; s__
4452700	1	3	3	1	3	1	4	7	3	3	11	8	2	18	5	11	8	13	13	7	k__Bacteria; p__Firmicutes; c__Clostridia; o__Clostridiales; f__Clostridiaceae; g__Clostridium; s__
592317	12	24	19	9	17	2	11	25	4	11	48	28	33	59	9	41	37	30	46	33	k__Bacteria; p__Firmicutes; c__Clostridia; o__Clostridiales; f__Clostridiaceae; g__Clostridium; s__
904668	12	25	20	14	24	4	14	41	16	7	40	40	24	55	9	65	45	34	42	31	k__Bacteria; p__Firmicutes; c__Clostridia; o__Clostridiales; f__Clostridiaceae; g__Clostridium; s__
818777	18	22	15	14	19	5	6	42	6	10	25	25	21	48	7	41	54	41	38	37	k__Bacteria; p__Firmicutes; c__Clostridia; o__Clostridiales; f__Clostridiaceae; g__Clostridium; s__
530061	24	17	20	8	33	5	17	30	22	21	37	41	34	71	12	45	48	31	66	32	k__Bacteria; p__Firmicutes; c__Clostridia; o__Clostridiales; f__Clostridiaceae; g__Clostridium; s__
523589	4	8	7	10	10	5	8	23	3	9	21	31	35	47	4	19	14	13	21	16	k__Bacteria; p__Firmicutes; c__Clostridia; o__Clostridiales; f__Clostridiaceae; g__Clostridium; s__neonatale
868555	3	2	1	2	1	1	4	7	2	2	2	2	5	2	4	4	3	6	3	8	k__Bacteria; p__Planctomycetes; c__Planctomycetia; o__Gemmatales; f__Isosphaeraceae; g__; s__
880673	5	3	1	1	8	3	2	3	7	5	2	3	2	8	2	8	7	4	7	3	k__Bacteria; p__Planctomycetes; c__Planctomycetia; o__Gemmatales; f__Isosphaeraceae; g__; s__
972396	6	8	7	7	11	5	7	7	14	2	6	13	11	4	8	8	7	8	5	9	k__Bacteria; p__Planctomycetes; c__Planctomycetia; o__Gemmatales; f__Isosphaeraceae; g__; s__
4474906	14	21	13	6	3	10	11	9	11	18	13	6	14	11	24	3	17	17	24	19	k__Bacteria; p__Planctomycetes; c__Planctomycetia; o__Gemmatales; f__Isosphaeraceae; g__; s__
213225	6	4	5	4	4	19	9	10	20	5	2	3	8	2	4	3	5	1	1	4	k__Bacteria; p__Planctomycetes; c__Planctomycetia; o__Pirellulales; f__Pirellulaceae; g__; s__
113272	306	253	244	148	298	4	66	81	26	27	131	85	164	127	15	104	60	46	56	73	k__Bacteria; p__Planctomycetes; c__Planctomycetia; o__Pirellulales; f__Pirellulaceae; g__; s__
590913	23	36	17	20	47	5	16	118	24	26	91	69	140	122	39	246	67	57	87	89	k__Bacteria; p__Planctomycetes; c__Planctomycetia; o__Pirellulales; f__Pirellulaceae; g__; s__
208621	1	3	2	2	2	14	23	28	31	28	3	2	1	2	4	6	14	17	9	19	k__Bacteria; p__Planctomycetes; c__Planctomycetia; o__Pirellulales; f__Pirellulaceae; g__A17; s__
222768	7	10	10	6	14	64	45	4	26	30	34	23	34	38	33	31	37	37	48	52	k__Bacteria; p__Proteobacteria; c__Alphaproteobacteria; o__; f__; g__; s__
564348	1	3	1	1	3	20	4	10	23	22	3	6	7	6	8	2	3	7	2	5	k__Bacteria; p__Proteobacteria; c__Alphaproteobacteria; o__Caulobacterales; f__Caulobacteraceae; g__; s__
589382	6	1	6	4	4	6	24	20	35	21	20	25	35	22	12	20	12	16	12	9	k__Bacteria; p__Proteobacteria; c__Alphaproteobacteria; o__Caulobacterales; f__Caulobacteraceae; g__; s__
751862	7	5	14	9	7	33	25	12	25	25	19	22	25	30	224	12	12	19	8	12	k__Bacteria; p__Proteobacteria; c__Alphaproteobacteria; o__Caulobacterales; f__Caulobacteraceae; g__; s__
288283	24	49	28	43	45	6	29	48	24	14	28	23	35	49	16	29	29	27	36	22	k__Bacteria; p__Proteobacteria; c__Alphaproteobacteria; o__Caulobacterales; f__Caulobacteraceae; g__; s__
242829	15	13	11	12	18	30	20	23	12	21	8	6	4	4	9	9	16	9	7	5	k__Bacteria; p__Proteobacteria; c__Alphaproteobacteria; o__Caulobacterales; f__Caulobacteraceae; g__Phenylobacterium; s__
417101	9	1	10	7	9	12	9	6	4	18	16	28	19	24	21	2	9	21	9	18	k__Bacteria; p__Proteobacteria; c__Alphaproteobacteria; o__Ellin329; f__; g__; s__
214914	2	4	2	1	4	33	23	45	35	26	13	7	6	5	4	33	17	18	13	32	k__Bacteria; p__Proteobacteria; c__Alphaproteobacteria; o__Ellin329; f__; g__; s__
555926	5	11	10	9	10	194	68	22	38	147	29	27	30	17	135	7	4	4	3	3	k__Bacteria; p__Proteobacteria; c__Alphaproteobacteria; o__Ellin329; f__; g__; s__
215825	13	7	5	6	28	12	69	20	20	28	145	98	106	151	216	3	9	25	17	15	k__Bacteria; p__Proteobacteria; c__Alphaproteobacteria; o__Ellin329; f__; g__; s__
2679706	3	5	5	5	5	107	23	42	24	92	5	21	15	10	64	71	67	68	115	105	k__Bacteria; p__Proteobacteria; c__Alphaproteobacteria; o__Ellin329; f__; g__; s__
211231	20	19	26	40	26	14	22	31	23	23	39	29	22	19	42	7	9	12	6	19	k__Bacteria; p__Proteobacteria; c__Alphaproteobacteria; o__Ellin329; f__; g__; s__
212223	25	37	38	18	56	192	166	75	233	122	46	38	41	56	96	84	98	116	73	108	k__Bacteria; p__Proteobacteria; c__Alphaproteobacteria; o__Ellin329; f__; g__; s__
1105814	190	283	187	157	167	333	212	217	181	264	435	398	407	316	791	264	240	243	173	162	k__Bacteria; p__Proteobacteria; c__Alphaproteobacteria; o__Rhizobiales; f__Bradyrhizobiaceae; g__Bradyrhizobium; s__
849118	12	13	22	15	13	37	53	33	103	61	48	53	30	16	90	26	23	31	29	38	k__Bacteria; p__Proteobacteria; c__Alphaproteobacteria; o__Rhizobiales; f__Hyphomicrobiaceae; g__Devosia; s__
1053775	32	29	24	21	33	20	17	19	24	21	36	67	27	24	42	32	28	24	31	16	k__Bacteria; p__Proteobacteria; c__Alphaproteobacteria; o__Rhizobiales; f__Hyphomicrobiaceae; g__Devosia; s__
244310	1	2	2	6	2	8	8	11	5	19	3	4	4	3	7	1	5	4	3	4	k__Bacteria; p__Proteobacteria; c__Alphaproteobacteria; o__Rhizobiales; f__Hyphomicrobiaceae; g__Rhodoplanes; s__
341831	12	4	24	9	19	203	81	33	37	56	11	11	17	1	24	23	69	71	28	96	k__Bacteria; p__Proteobacteria; c__Alphaproteobacteria; o__Rhizobiales; f__Hyphomicrobiaceae; g__Rhodoplanes; s__
4402900	6	1	15	3	1	15	4	2	1	6	2	11	10	2	25	3	4	12	6	12	k__Bacteria; p__Proteobacteria; c__Alphaproteobacteria; o__Rhizobiales; f__Hyphomicrobiaceae; g__Rhodoplanes; s__
545247	3	4	2	1	7	13	2	5	6	1	2	4	2	1	18	9	6	6	7	14	k__Bacteria; p__Proteobacteria; c__Alphaproteobacteria; o__Rhizobiales; f__Hyphomicrobiaceae; g__Rhodoplanes; s__
222350	2	3	4	6	2	3	14	13	28	30	6	4	5	1	21	3	13	25	15	19	k__Bacteria; p__Proteobacteria; c__Alphaproteobacteria; o__Rhizobiales; f__Hyphomicrobiaceae; g__Rhodoplanes; s__
541612	68	72	68	36	62	6	1	5	4	5	27	15	18	21	35	1	3	6	4	2	k__Bacteria; p__Proteobacteria; c__Alphaproteobacteria; o__Rhizobiales; f__Hyphomicrobiaceae; g__Rhodoplanes; s__
511627	5	2	9	2	1	1	11	16	22	11	3	10	5	4	19	1	1	4	2	5	k__Bacteria; p__Proteobacteria; c__Alphaproteobacteria; o__Rhizobiales; f__Hyphomicrobiaceae; g__Rhodoplanes; s__
555387	9	11	15	5	37	27	35	48	60	26	79	118	60	64	367	34	17	20	22	14	k__Bacteria; p__Proteobacteria; c__Alphaproteobacteria; o__Rhizobiales; f__Hyphomicrobiaceae; g__Rhodoplanes; s__
4371078	28	10	36	11	13	102	65	43	160	118	26	53	57	25	130	69	61	75	77	69	k__Bacteria; p__Proteobacteria; c__Alphaproteobacteria; o__Rhizobiales; f__Hyphomicrobiaceae; g__Rhodoplanes; s__
319907	22	11	11	13	15	78	65	36	94	68	28	55	46	19	232	10	25	32	23	53	k__Bacteria; p__Proteobacteria; c__Alphaproteobacteria; o__Rhizobiales; f__Hyphomicrobiaceae; g__Rhodoplanes; s__
562311	156	104	143	119	89	13	44	54	106	42	74	64	60	119	80	105	57	76	91	60	k__Bacteria; p__Proteobacteria; c__Alphaproteobacteria; o__Rhizobiales; f__Hyphomicrobiaceae; g__Rhodoplanes; s__
557209	14	7	12	25	11	11	21	3	15	16	18	22	25	14	98	27	21	38	21	30	k__Bacteria; p__Proteobacteria; c__Alphaproteobacteria; o__Rhizobiales; f__Methylocystaceae; g__; s__
3798902	48	50	40	42	54	27	15	8	18	11	39	48	42	47	93	70	31	39	36	26	k__Bacteria; p__Proteobacteria; c__Alphaproteobacteria; o__Rhizobiales; f__Methylocystaceae; g__; s__
4322256	6	2	8	4	5	3	8	2	10	5	6	5	5	2	8	6	8	7	6	2	k__Bacteria; p__Proteobacteria; c__Alphaproteobacteria; o__Rhodospirillales; f__Acetobacteraceae; g__; s__
722618	10	5	4	5	5	23	9	15	32	19	13	14	16	6	59	6	23	23	19	31	k__Bacteria; p__Proteobacteria; c__Alphaproteobacteria; o__Rhodospirillales; f__Acetobacteraceae; g__; s__
568243	177	51	139	78	53	14	10	9	11	13	38	47	40	16	27	8	15	11	6	8	k__Bacteria; p__Proteobacteria; c__Alphaproteobacteria; o__Rhodospirillales; f__Acetobacteraceae; g__; s__
271114	16	5	6	6	11	233	17	13	7	54	3	16	6	4	9	17	25	26	24	17	k__Bacteria; p__Proteobacteria; c__Alphaproteobacteria; o__Rhodospirillales; f__Acetobacteraceae; g__Acidocella; s__
4439343	2	9	5	2	3	6	11	5	43	16	13	11	18	7	24	2	1	2	3	5	k__Bacteria; p__Proteobacteria; c__Alphaproteobacteria; o__Rhodospirillales; f__Rhodospirillaceae; g__; s__
262768	1	1	4	2	4	41	27	24	5	30	1	11	7	3	18	17	14	50	13	42	k__Bacteria; p__Proteobacteria; c__Alphaproteobacteria; o__Rhodospirillales; f__Rhodospirillaceae; g__; s__
222088	3	4	3	2	3	21	8	4	4	17	3	3	2	5	8	3	3	10	8	15	k__Bacteria; p__Proteobacteria; c__Alphaproteobacteria; o__Rhodospirillales; f__Rhodospirillaceae; g__; s__
114130	12	9	5	7	21	71	98	67	154	67	52	15	25	17	163	50	34	43	80	63	k__Bacteria; p__Proteobacteria; c__Alphaproteobacteria; o__Rhodospirillales; f__Rhodospirillaceae; g__; s__
549969	111	121	89	65	108	7	20	9	25	9	71	45	61	67	55	155	127	94	110	90	k__Bacteria; p__Proteobacteria; c__Alphaproteobacteria; o__Rhodospirillales; f__Rhodospirillaceae; g__; s__
564337	131	68	112	101	46	8	24	39	26	20	128	151	142	166	53	45	55	58	32	26	k__Bacteria; p__Proteobacteria; c__Alphaproteobacteria; o__Rhodospirillales; f__Rhodospirillaceae; g__; s__
564004	42	38	46	47	53	58	37	18	34	31	18	32	25	12	178	11	30	38	19	55	k__Bacteria; p__Proteobacteria; c__Alphaproteobacteria; o__Rhodospirillales; f__Rhodospirillaceae; g__; s__
1082059	2	3	5	2	1	12	1	3	1	4	4	6	7	2	12	4	3	6	8	9	k__Bacteria; p__Proteobacteria; c__Alphaproteobacteria; o__Sphingomonadales; f__Erythrobacteraceae; g__Erythrobacter; s__nanhaisediminis
1052559	11	8	11	9	11	4	3	6	1	9	7	3	2	3	7	1	3	5	2	3	k__Bacteria; p__Proteobacteria; c__Alphaproteobacteria; o__Sphingomonadales; f__Sphingomonadaceae; g__; s__
699318	10	2	4	16	7	102	53	34	17	64	5	7	19	3	14	8	23	107	10	40	k__Bacteria; p__Proteobacteria; c__Alphaproteobacteria; o__Sphingomonadales; f__Sphingomonadaceae; g__; s__
754410	17	7	21	18	14	69	33	27	10	33	47	90	96	28	252	7	17	71	20	47	k__Bacteria; p__Proteobacteria; c__Alphaproteobacteria; o__Sphingomonadales; f__Sphingomonadaceae; g__; s__
4369884	6	4	13	2	4	1	9	18	4	4	15	31	24	13	16	2	10	7	6	2	k__Bacteria; p__Proteobacteria; c__Alphaproteobacteria; o__Sphingomonadales; f__Sphingomonadaceae; g__Kaistobacter; s__
849863	2	2	2	2	1	2	3	2	1	4	2	1	2	1	5	3	1	2	3	6	k__Bacteria; p__Proteobacteria; c__Alphaproteobacteria; o__Sphingomonadales; f__Sphingomonadaceae; g__Kaistobacter; s__
1121270	6	3	6	7	3	5	8	12	2	9	4	3	4	2	11	7	3	4	2	10	k__Bacteria; p__Proteobacteria; c__Alphaproteobacteria; o__Sphingomonadales; f__Sphingomonadaceae; g__Kaistobacter; s__
989109	29	12	13	48	28	54	55	47	13	61	14	17	25	9	56	3	19	28	12	29	k__Bacteria; p__Proteobacteria; c__Alphaproteobacteria; o__Sphingomonadales; f__Sphingomonadaceae; g__Kaistobacter; s__
949062	34	12	14	37	23	18	29	19	3	33	7	8	7	12	18	7	8	16	3	10	k__Bacteria; p__Proteobacteria; c__Alphaproteobacteria; o__Sphingomonadales; f__Sphingomonadaceae; g__Kaistobacter; s__
4453972	29	42	33	34	57	3	34	107	7	47	31	22	31	41	36	314	90	103	152	110	k__Bacteria; p__Proteobacteria; c__Alphaproteobacteria; o__Sphingomonadales; f__Sphingomonadaceae; g__Kaistobacter; s__
2294362	46	24	43	48	19	102	141	151	68	115	102	70	76	96	180	15	49	50	28	60	k__Bacteria; p__Proteobacteria; c__Alphaproteobacteria; o__Sphingomonadales; f__Sphingomonadaceae; g__Kaistobacter; s__
654742	240	188	224	230	249	477	390	504	82	373	287	201	257	142	661	173	199	316	182	232	k__Bacteria; p__Proteobacteria; c__Alphaproteobacteria; o__Sphingomonadales; f__Sphingomonadaceae; g__Kaistobacter; s__
1108960	15	7	28	12	15	3	2	2	2	8	24	5	23	61	1	9	14	12	24	17	k__Bacteria; p__Proteobacteria; c__Alphaproteobacteria; o__Sphingomonadales; f__Sphingomonadaceae; g__Sphingomonas; s__
647775	37	48	28	37	50	19	22	29	24	24	22	43	18	42	16	16	21	15	38	25	k__Bacteria; p__Proteobacteria; c__Betaproteobacteria; o__Burkholderiales; f__Comamonadaceae; g__; s__
4341348	4	3	4	4	1	3	40	36	54	16	3	2	2	2	6	3	3	4	2	2	k__Bacteria; p__Proteobacteria; c__Betaproteobacteria; o__SC-I-84; f__; g__; s__
1623482	7	19	13	12	19	23	16	15	79	21	24	42	34	44	120	51	17	24	21	25	k__Bacteria; p__Proteobacteria; c__Betaproteobacteria; o__SC-I-84; f__; g__; s__
819893	15	11	16	30	21	116	88	85	117	52	15	43	21	8	120	7	19	34	23	23	k__Bacteria; p__Proteobacteria; c__Betaproteobacteria; o__SC-I-84; f__; g__; s__
540592	4	13	5	7	10	5	7	8	4	3	21	11	16	18	41	6	3	6	1	10	k__Bacteria; p__Proteobacteria; c__Deltaproteobacteria; o__Myxococcales; f__; g__; s__
4302753	3	2	2	1	1	37	24	6	9	14	1	2	1	1	2	5	9	2	2	7	k__Bacteria; p__Proteobacteria; c__Deltaproteobacteria; o__Myxococcales; f__; g__; s__
1052235	9	5	10	1	11	25	10	4	6	9	8	9	6	4	30	9	4	5	7	15	k__Bacteria; p__Proteobacteria; c__Deltaproteobacteria; o__Myxococcales; f__Haliangiaceae; g__; s__
388763	7	7	7	15	13	4	11	16	5	2	8	2	14	16	2	1	11	7	4	12	k__Bacteria; p__Proteobacteria; c__Gammaproteobacteria; o__Pseudomonadales; f__Pseudomonadaceae; g__Pseudomonas; s__
818602	269	271	285	207	308	122	124	251	175	101	184	169	157	255	62	139	193	137	226	138	k__Bacteria; p__Proteobacteria; c__Gammaproteobacteria; o__Pseudomonadales; f__Pseudomonadaceae; g__Pseudomonas; s__
252012	18	10	13	12	12	1	9	14	12	2	23	19	26	20	72	11	10	12	12	5	k__Bacteria; p__Proteobacteria; c__Gammaproteobacteria; o__Xanthomonadales; f__Sinobacteraceae; g__; s__
112168	215	112	147	122	136	1	60	90	62	62	394	293	386	356	193	145	141	124	139	85	k__Bacteria; p__Proteobacteria; c__Gammaproteobacteria; o__Xanthomonadales; f__Sinobacteraceae; g__; s__
366851	9	4	10	5	1	2	11	22	3	10	20	23	10	20	21	2	8	1	3	2	k__Bacteria; p__Proteobacteria; c__Gammaproteobacteria; o__Xanthomonadales; f__Sinobacteraceae; g__; s__
4354744	3	5	5	8	8	2	5	4	7	2	8	2	6	2	2	4	1	2	1	2	k__Bacteria; p__Proteobacteria; c__Gammaproteobacteria; o__Xanthomonadales; f__Sinobacteraceae; g__; s__
727692	7	7	5	7	6	2	1	5	4	5	5	10	5	4	9	3	1	4	11	1	k__Bacteria; p__Proteobacteria; c__Gammaproteobacteria; o__Xanthomonadales; f__Sinobacteraceae; g__; s__
1110303	87	86	106	88	109	3	19	14	7	12	77	75	96	118	62	84	45	46	65	30	k__Bacteria; p__Proteobacteria; c__Gammaproteobacteria; o__Xanthomonadales; f__Sinobacteraceae; g__; s__
111642	62	70	87	59	36	6	21	33	16	17	80	94	70	102	26	253	115	104	149	69	k__Bacteria; p__Proteobacteria; c__Gammaproteobacteria; o__Xanthomonadales; f__Sinobacteraceae; g__; s__
572643	57	56	69	73	126	14	31	42	36	27	77	33	55	78	75	44	15	19	24	12	k__Bacteria; p__Proteobacteria; c__Gammaproteobacteria; o__Xanthomonadales; f__Sinobacteraceae; g__; s__
509622	142	68	118	77	78	37	49	54	48	50	145	171	125	178	36	310	149	164	207	98	k__Bacteria; p__Proteobacteria; c__Gammaproteobacteria; o__Xanthomonadales; f__Sinobacteraceae; g__; s__
105699	2	1	34	9	13	41	87	22	81	29	18	9	11	5	18	87	101	73	83	171	k__Bacteria; p__Proteobacteria; c__Gammaproteobacteria; o__Xanthomonadales; f__Xanthomonadaceae; g__; s__
816868	24	2	13	7	15	2	10	3	20	2	2	2	7	8	1	23	32	14	28	38	k__Bacteria; p__Proteobacteria; c__Gammaproteobacteria; o__Xanthomonadales; f__Xanthomonadaceae; g__; s__
276133	3	1	4	5	7	15	12	7	7	91	1	4	3	3	1	3	6	132	4	6	k__Bacteria; p__Proteobacteria; c__Gammaproteobacteria; o__Xanthomonadales; f__Xanthomonadaceae; g__; s__
741918	248	997	326	195	573	6	55	21	113	44	200	153	174	246	75	90	146	106	185	149	k__Bacteria; p__Proteobacteria; c__Gammaproteobacteria; o__Xanthomonadales; f__Xanthomonadaceae; g__; s__
81089	868	1787	932	555	1376	32	71	27	62	42	609	462	679	806	180	453	341	311	593	369	k__Bacteria; p__Proteobacteria; c__Gammaproteobacteria; o__Xanthomonadales; f__Xanthomonadaceae; g__; s__
614944	1	2	3	2	4	15	4	2	2	33	2	6	1	1	1	4	3	72	3	9	k__Bacteria; p__Proteobacteria; c__Gammaproteobacteria; o__Xanthomonadales; f__Xanthomonadaceae; g__Fulvimonas; s__soli
513602	16	11	15	17	23	31	180	13	438	17	11	26	50	22	39	8	2	4	3	5	k__Bacteria; p__Proteobacteria; c__Gammaproteobacteria; o__Xanthomonadales; f__Xanthomonadaceae; g__Rhodanobacter; s__
3738429	4	26	11	1	7	10	6	13	10	13	23	24	38	13	51	23	26	16	19	14	k__Bacteria; p__Verrucomicrobia; c__[Pedosphaerae]; o__[Pedosphaerales]; f__; g__; s__
810360	1	9	2	2	10	26	3	9	9	27	23	11	17	8	10	9	5	4	30	7	k__Bacteria; p__Verrucomicrobia; c__[Pedosphaerae]; o__[Pedosphaerales]; f__auto67_4W; g__; s__
161263	25	25	37	42	23	8	18	22	58	16	150	84	165	73	9	2	5	3	6	3	k__Bacteria; p__Verrucomicrobia; c__[Pedosphaerae]; o__[Pedosphaerales]; f__auto67_4W; g__; s__
1108431	1	2	2	5	6	39	12	15	15	22	7	2	4	2	5	48	26	20	31	48	k__Bacteria; p__Verrucomicrobia; c__[Pedosphaerae]; o__[Pedosphaerales]; f__Ellin515; g__; s__
4348899	15	39	8	26	41	68	4	16	2	28	4	18	3	10	1	2	4	2	5	3	k__Bacteria; p__Verrucomicrobia; c__Opitutae; o__Opitutales; f__Opitutaceae; g__Opitutus; s__
156210	3	3	5	2	2	14	18	25	14	29	8	6	8	6	2	14	12	11	24	32	k__Bacteria; p__Verrucomicrobia; c__Opitutae; o__Opitutales; f__Opitutaceae; g__Opitutus; s__
797182	16	4	26	23	7	1	11	15	9	18	5	6	26	4	1	6	37	41	17	15	k__Bacteria; p__WPS-2; c__; o__; f__; g__; s__
1057310	72	42	58	52	64	4	28	56	12	35	59	46	58	78	15	129	85	75	99	76	k__Bacteria; p__WPS-2; c__; o__; f__; g__; s__
1059748	322	177	258	172	103	7	148	201	175	145	117	101	136	104	29	107	352	222	144	131	k__Bacteria; p__WPS-2; c__; o__; f__; g__; s__
1135287	168	67	165	67	54	7	166	162	226	150	133	103	157	136	33	197	413	294	256	183	k__Bacteria; p__WPS-2; c__; o__; f__; g__; s__
246196	8	4	4	7	5	115	175	110	195	91	28	27	26	5	49	36	96	77	70	177	k__Bacteria; p__Gemmatimonadetes; c__Gemmatimonadetes; o__Ellin5290; f__; g__; s__
109789	12	7	10	9	9	1	4	2	10	2	12	15	18	25	2	5	1	2	8	3	k__Bacteria; p__Nitrospirae; c__Nitrospira; o__Nitrospirales; f__Nitrospiraceae; g__Nitrospira; s__
111162	5	11	9	5	8	22	15	34	10	37	13	22	15	6	48	18	21	9	10	36	k__Bacteria; p__Planctomycetes; c__Phycisphaerae; o__WD2101; f__; g__; s__
225556	1	12	7	2	3	2	33	45	25	29	15	4	4	10	1	15	12	14	24	13	k__Bacteria; p__AD3; c__ABS-6; o__; f__; g__; s__
4390608	38	20	51	47	25	3	2	3	1	1	12	25	16	26	11	3	2	4	6	2	k__Bacteria; p__Bacteroidetes; c__Cytophagia; o__Cytophagales; f__Cytophagaceae; g__Sporocytophaga; s__
